# The histamine H3 receptor modulates dopamine D2 receptor–dependent signaling pathways and mouse behaviors

**DOI:** 10.1016/j.jbc.2023.104583

**Published:** 2023-03-04

**Authors:** Jian Xu, Christopher Pittenger

**Affiliations:** 1 Department of Psychiatry, Yale University; 2 Department of Psychology, Yale University; 3 Department of Child Study Center, Yale University; 4 Department of Interdepartmental Neuroscience Program, Yale University; 5 Department of Wu-Tsai Institute, Yale University; 6 Department of Center for Brain and Mind Health, Yale University

**Keywords:** dopamine receptor, D2R, G protein–coupled receptor, histamine, H3R, locomotor activity, phosphorylation, protein complex, signaling transduction, spiny projection neurons

## Abstract

The histamine H3 receptor (H3R) is highly enriched in the spiny projection neurons (SPNs) of the striatum, in both the D1 receptor (D1R)–expressing and D2 receptor (D2R)–expressing populations. A crossantagonistic interaction between H3R and D1R has been demonstrated in mice, both at the behavioral level and at the biochemical level. Although interactive behavioral effects have been described upon coactivation of H3R and D2R, the molecular mechanisms underlying this interaction are poorly understood. Here, we show that activation of H3R with the selective agonist R-(−)-α-methylhistamine dihydrobromide mitigates D2R agonist–induced locomotor activity and stereotypic behavior. Using biochemical approaches and the proximity ligation assay, we demonstrated the existence of an H3R–D2R complex in the mouse striatum. In addition, we examined consequences of simultaneous H3R–D2R agonism on the phosphorylation levels of several signaling molecules using immunohistochemistry. H3R agonist treatment modulated Akt (serine/threonine PKB)–glycogen synthase kinase 3 beta signaling in response to D2R activation *via* a β-arrestin 2–dependent mechanism in D2R-SPNs but not in D1R-SPNs. Phosphorylation of mitogen- and stress-activated protein kinase 1 and rpS6 (ribosomal protein S6) was largely unchanged under these conditions. As Akt–glycogen synthase kinase 3 beta signaling has been implicated in several neuropsychiatric disorders, this work may help clarify the role of H3R in modulating D2R function, leading to a better understanding of pathophysiology involving the interaction between histamine and dopamine systems.

The striatum, which is subdivided in primates into the caudate and putamen, is the major input nucleus of the basal ganglia and integrates synaptic inputs from cortical and thalamic afferents (1). It plays critical roles in motor coordination, reward-driven learning, goal-directed behaviors, habitual behaviors, and other processes (2, 3, 4, 5). Disruption of basal ganglia function has been implicated in a wide range of neuropsychiatric disorders, including Tourette syndrome (TS) and other tic disorders, obsessive compulsive disorder, drug abuse, and many other conditions (6, 7, 8). Function of the basal ganglia circuitry is modulated by many neurotransmitters, including glutamate, dopamine (DA), γ-aminobutyric acid (GABA), and acetylcholine (9, 10).

The neurotransmitter histamine (HA) has been more recently shown to critically regulate basal ganglia function (11, 12, 13). HA is produced by neurons in the posterior tuberomamillary nucleus of the hypothalamus (14, 15, 16). The effects of HA are mediated by four HA receptors, H1R, H2R, H3R, and H4R (14, 17). These G protein–coupled 7-transmembrane proteins work through several downstream signaling pathways, including both cAMP-dependent and cAMP-independent mechanisms (17).

The H3R is of particular interest. It is highly expressed in the striatum (14, 15) and has several very interesting characteristics, including the presence of many isoforms, constitutive activity even in the absence of ligand, and both presynaptic and postsynaptic localization (17, 18, 19). H3R has traditionally been thought to act presynaptically to regulate the release of both HA and other neurotransmitters (14, 20). However, it is increasingly clear that much of the H3R in the striatum is localized postsynaptically on spiny projection neurons (SPNs), and that this postsynaptic H3R can importantly regulate striatal function (16, 17, 21).

Recent work has revealed complex interactions between histaminergic and dopaminergic modulation of the basal ganglia. A rare mutation in histidine decarboxylase (*Hdc*), the biosynthetic enzyme required for HA production, has been associated with TS (22). Elevated striatal DA is observed in a mouse model of *Hdc* deficiency, which displays several behavioral phenotypes that may recapitulate symptoms of TS (23, 24). Behavioral abnormalities in this mouse are reversed by the D2R antagonist haloperidol. At the cellular level, HA–DA interaction is likely to take place in the GABAergic principle neurons in the striatum, the SPNs (also known as medium spiny neurons [MSNs]), which make up 90 to 95% of striatal neurons. SPNs are morphologically homogeneous but consist of two subpopulations distinguished by their DA receptor expression and primary projection target: the D1 receptor (D1R)–expressing SPNs of the “direct pathway” and the D2 receptor (D2R)–expressing SPNs of the “indirect pathway” (1, 25). H3R is expressed in both D1R- and D2R-SPNs at the level of both mRNA and protein (26, 27).

It has been proposed that G protein–coupled receptors (GPCRs) can form homomers and heteromers, and that heterodimerization modulates receptor function and downstream signaling (21, 27, 28, 29, 30, 31). While heteromerization of class C GPCRs (such as taste receptors and metabotropic glutamate receptors, which are obligate dimers) is generally accepted, heteromerization of class A GPCRs (such as DA, adenosine, and HA receptors) is less well established (32, 33, 34). Visualizing higher-order endogenous receptor complexes and distinguishing between physical and functional interactions with confidence is technically challenging. Monomeric class A GPCRs have been shown to be sufficient to initiate downstream signaling in some contexts (35, 36, 37). However, accumulating evidence suggests that class A GPCRs do heterodimerize under some circumstances (31, 38, 39).

H3R can form heteromers with both D1R and D2R in reduced systems (21, 40). Most *in vivo* work has focused on the functional consequences of H3R–D1R interactions, on both behavioral effects and downstream signaling (27, 40, 41). A crossantagonism model of H3R–D1R interactions has been proposed: both H3R and D1R agonists activate mitogen-activated protein kinase (MAPK) signaling when used alone, but coadministration of an H3R agonist or antagonist blocks D1R-induced MAPK signaling (27, 42). We have extended these findings *in vivo*, showing that H3R–D1R interaction modulates MSK1 (mitogen- and stress-activated protein kinase 1, a downstream target of MAPK) and rpS6 (ribosomal protein S6) in D1R-SPNs *in vivo* in the mouse striatum (41). We and others have also shown functional interactions between H3R and D1R effects on mouse behavior, using locomotor activity as a readout (41).

Much less work has examined H3R–D2R interactions. H3R and D2R agonists have interactive effects on locomotor behavior in reserpinized mice (21), but the molecular correlates of this interaction have not been identified. In this study, we examine functional and physical interactions between H3R and D2R in D2R-SPNs using behavioral tests, immunohistochemical staining with automated imaging processing, a proximity ligation assay (PLA), and biochemical approaches. We confirm the expression of H3R in both D1R- and D2R-SPNs (15, 26, 27) and demonstrate that they associate in a complex using coimmunoprecipitation, in agreement with a previous report (27). We also confirm that H3R localizes in close proximity with both D2R and D1R using a PLA in mouse striatal slices. At the behavioral level, coactivation of H3R using the selective agonist R-(−)-α-methylhistamine dihydrobromide (RAMH) attenuated locomotion and stereotypy produced by the D2R-agonist quinpirole (Quin; in reserpinized mice) and the D1R/D2R dual agonist apomorphine (Apo; in intact mice). At the signaling level, we observed functional interactions of H3R and D2R agonists on the regulation of the Akt (serine/threonine PKB)–glycogen synthase kinase 3 beta (GSK3β) signaling pathway in D2R-SPNs but not in D1R-SPNs. We have previously shown that the MAPK signaling is preferentially regulated by H3R and D1R in D1R-SPNs but not in D2R-SPNs; in conjunction with the current results, this reveals complex differential signaling effects of HA in the striatum.

## Results

### Coactivation of HA H3 receptor attenuates DA D2 receptor agonist–induced locomotor and stereotypic behavior in mice

The direct application of D2R agonists, such as Quin, induces mixed effects on behavior because of the localization of D2R at both postsynaptic and presynaptic sites (14, 16). Some studies have described biphasic effects of Quin on locomotor activity (43, 44). To overcome this complexity, many studies use depletion of presynaptic DA (and other neurotransmitters) to isolate the effects of postsynaptic D2R signaling. A single reserpine injection (1–5 mg/kg) blocks the vesicular monoamine transporter and causes a long-lasting >90% reduction in striatal DA concentration in rats (45). These effects persist for up to 30 days (46). We performed a pilot study and determined that 2 mg/kg reserpine injection (s.c.) 20 h prior to other drug treatments worked well in our hands in mice.

To allow subsequent identification of D1R- and D2R-expressing SPNs using immunochemistry, all behavioral tests were carried out in dopamine- and cAMP-regulated phosphoprotein of molecular weight 32 kDa (DARPP-32)–tagged double transgenic mice, referred to as D1-FLAG/D2-Myc mice (47). Because of the limited availability of these mice when we started this work, we used a crossover design in which all mice (9 males and 16 females) received all four treatment combinations (RAMH *versus* saline [Sal], Quin *versus* Sal) in counterbalanced order across four consecutive days and then monitored in an open field. Baseline activity (locomotion and stereotypy in the open field) increased over the course of these 4 days, as the effect of reserpine began to wear off, although baseline activity remained an order of magnitude lower than that seen in nonreserpinized mice even 4 days after reserpine (not shown). To account for both repeated measures and this baseline drift, data were analyzed using a linear mixed effects model with daily baseline activity as a covariate, Quin *versus* Sal and RAMH *versus* Sal as within-subject factors, sex as an independent factor, and animal as a random variable. Covariate-adjusted values are shown here to isolate drug effects; raw activity values are shown in Fig. S1.

**Figure 1:**
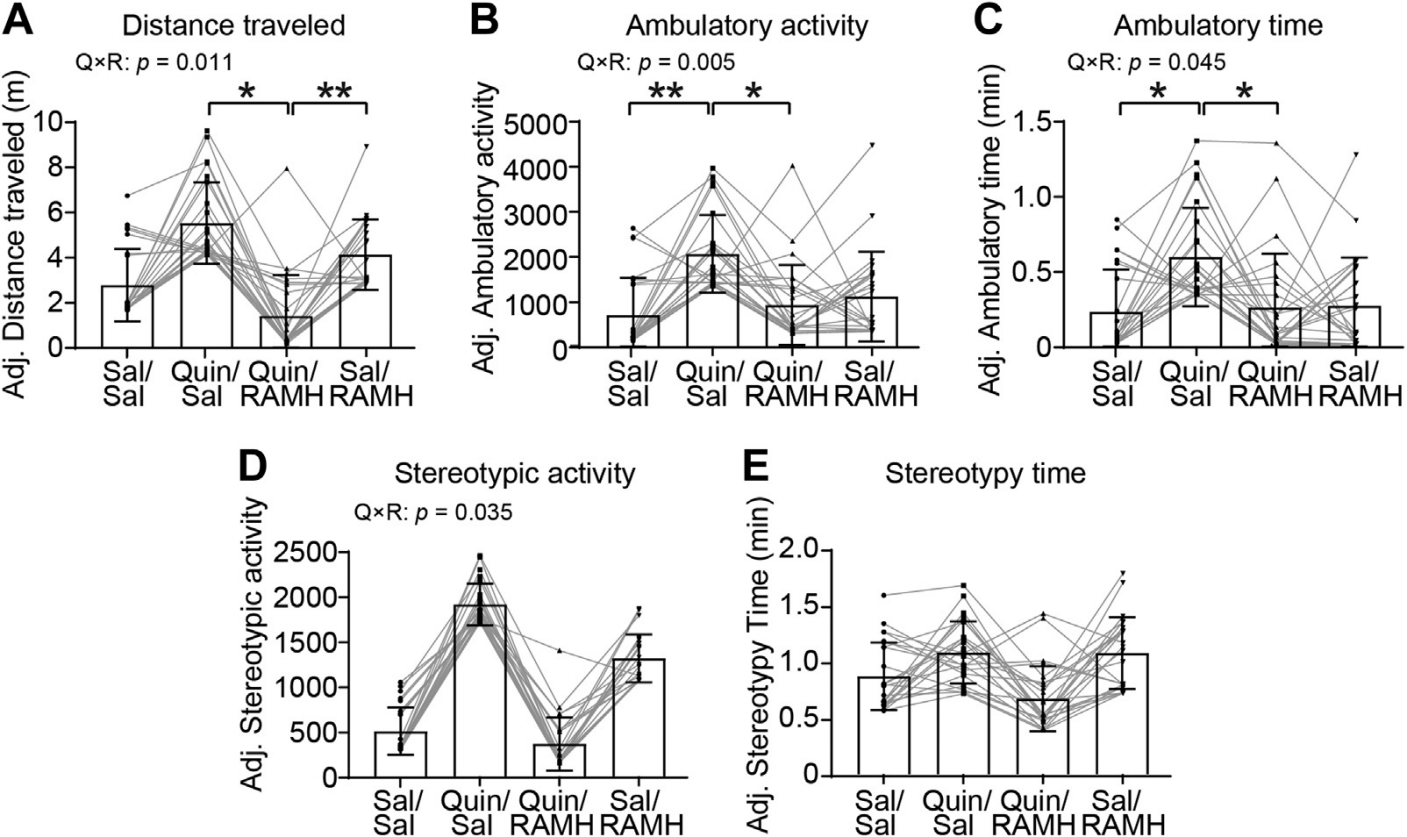
**H3R agonist coadministration attenuates D2R agonist-induced locomotor and stereotypic behavior.** Male and female D1-FLAG/D2-Myc mice received reserpine (2 mg/kg, s.c.) 20 h prior to the first drug administration. Mice were placed in activity chambers for 30 min, received injections of saline (Sal) or RAMH (45 mg/kg, i.p.), followed by Sal or quinpirole (Quin, 0.5 mg/kg, i.p.), and then monitored for 45 min. Covariate-adjusted values are plotted for clearer drug effects; statistical analysis was performed using raw values. *A*, distance traveled. Quin × RAMH interaction, *F*(1, 31.36) = 7.351, *p* = 0.011. *B*, ambulatory activity counts. Quin × RAMH interaction: *F*(1, 33.00) = 9.071, *p* = 0.005; main effect of Quin: *F*(1, 35.81) = 5.092, *p* = 0.030. *C*, ambulatory activity time. Quin × RAMH interaction: *F*(1, 25.70) = 4.424, *p* = 0.045. *D*, stereotypic activity counts. Quin × RAMH interaction: *F*(1, 40.37) = 4.750, *p* = 0.035. *E*, stereotypy time. Quin × RAMH interaction: *F*(1, 41.39) = 1.046, *p* = 0.312. All values are expressed as mean ± SD. Statistical analysis was performed using a linear mixed effects model with baseline activity as a covariate in SPSS 28. See Table S2 for additional statistical details. Where significant drug interactions or main effects were detected, multiple comparisons were conducted using post hoc Bonferroni test. ∗*p* < 0.05, ∗∗*p* < 0.01, n = 24 each group (9 male and 15 female mice). D2R, dopamine 2 receptor; H3R, histamine H3 receptor; RAMH, R-(−)-α-methylhistamine dihydrobromide.

We first examined the effect of H3R and D2R agonism on distance traveled in the open field. No sex differences were observed when sex was included as an independent factor (not shown), so data from males and females were combined. There was a significant interaction between RAMH and Quin (*p* = 0.011) in total distance traveled (Fig. 1*A*). Post hoc pairwise comparisons (Bonferroni corrected) revealed that Quin treatment induced a trend-level increase in the distance traveled relative to Sal treatment (*p* = 0.164). Coadministration of RAMH attenuated the locomotor effect by Quin (Quin/Sal *versus* Quin/RAMH: *p* = 0.032). Coactivation of H3R and D2R also produced lower locomotor activity than did H3R activation alone by RAMH (*p* = 0.002). Similar effects were seen in ambulatory activity (RAMH × Quin interaction: *p* = 0.005; Fig. 1*B*) and time spent in ambulation (interaction: *p* = 0.045; Fig. 1*C*). Pairwise post hoc comparisons showed that Quin significantly increased ambulatory activity (*p* = 0.006) and ambulatory time (*p* = 0.050) relative to the Sal. Coadministration of RAMH blocked Quin-induced activity (*p* = 0.018, Fig. 1*B*; *p* = 0.044, Fig. 1*C*).

In addition to locomotor effects, activation of D2R has also been shown to induce stereotypic behaviors (48). We examined stereotypic activity counts (Fig. 1*D*) and time spent in stereotypy (Fig. 1*E*). A significant Quin × RAMH interaction was found in stereotypic activity counts (*p* = 0.035) but not in stereotypy time (*p* = 0.312). Post hoc pairwise comparisons revealed that Quin induced a trend-level increase in stereotypic activity relative to the control group (*p* = 0.111), which was reversed, again at trend level, by cotreatment of RAMH (Quin/Sal *versus* Quin/RAMH, *p* = 0.079).

### Expression of H3R in D1R- and D2R-SPNs

Previous findings have shown that the H3R is expressed in both D1R- and D2R-SPNs in the striatum using immunostaining (26, 27). To validate the presence of H3R in these cell types, we also examined Hrh3 gene expression in several cell types in the mouse striatum. We searched publicly available datasets obtained by single-cell RNA sequencing, which allows the generation of high-throughput gene expression data at single-cell resolution. Among the cell types clustered, Hrh3 is found to be highly expressed in both D1- and D2-SPNs, together with some interneuron types (49, 50, 51, 52) (Table S3), matching the distribution of H3R in D1- and D2-SPNs.

**Figure 2:**
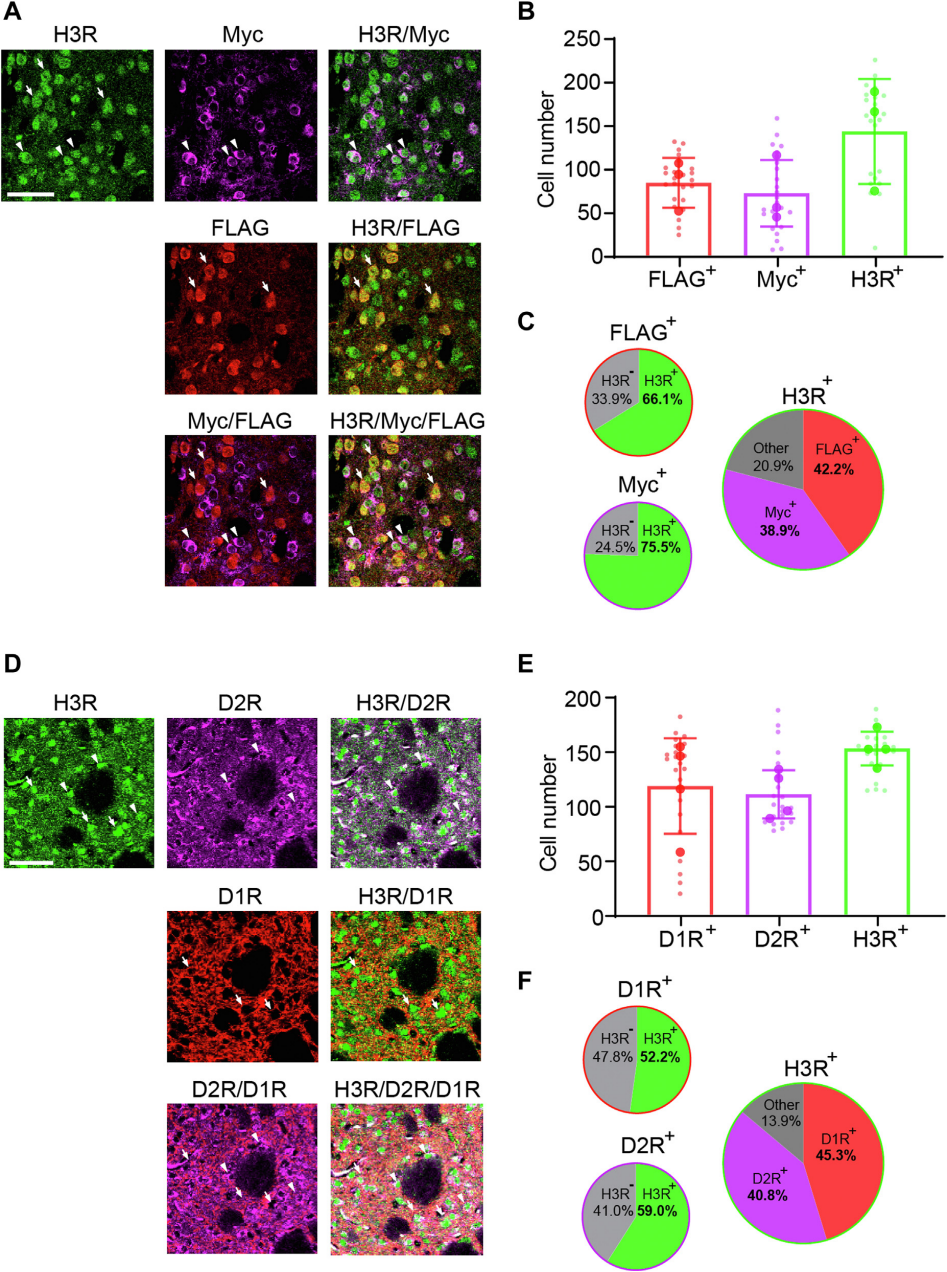
**H3R expression in both D1R- and D2R-SPNs.** Striatal sections from male and female D1-FLAG/D2-Myc mice (*A*–*C*) and C57BL/6J mice (*D*–*F*) were used for triple immunostaining. *A*, representative images of H3R immunostaining (*green*) in D1R- and D2R-SPNs. *White arrows* indicate D1R-SPNs (labeled by the FLAG epitope in *red*), whereas *arrowheads* indicate D2R-SPNs (labeled by the Myc epitope in *magenta*). Merged images were obtained by overlaying channels from the same field of view as indicated. Scale bar represents 50 μm. *B*, cell counts by different markers. *C*, proportions of cells that coexpressed two markers. *D*, representative images of H3R immunostaining (*green*) in D1R- and D2R-SPNs. *White arrows* indicate D1R immunoreactivity (labeled by anti-D1R antibody in *red*), whereas *arrowheads* indicate D2R immunoreactivity (labeled by the anti-D2R antibody in *magenta*). Merged images were obtained by overlaying channels from the same field of view as indicated. Scale bar represents 50 μm. *E*, cell counts by different markers. *F*, proportions of cells that coexpressed two markers. Values in *B* and *F* are expressed as mean ± SD. n = 3 for *A–C* and n = 4 for *D* and *E*. D1R, dopamine 1 receptor; D2R, dopamine 2 receptor; H3R, histamine H3 receptor; SPN, spiny projection neuron.

To confirm the expression of H3R in both D1- and D2-SPNs, we took advantage of the D1-FLAG/D2-Myc mice, which allowed easy and highly specific labeling of D1R- and D2R-SPNs using different antigen epitopes (47). Striatal brain sections were triple-immunostained with anti-H3R, anti-FLAG (for D1 cells), and anti-Myc (for D2 cells) antibodies. Cells labeled by each of the antibodies, visualized in separate channels in confocal images, were identified using an object-based approach (Fig. 2, *A–C*). Overlapping objects were compared between channels. We found that 66.1% FLAG-positive cells (D1R-SPNs) and 75.5% Myc-positive cells (D2-SPNs) also coexpressed H3R. In the converse analysis, 42.2% and 38.9% of H3R-positive cells expressed FLAG (D1R) and Myc (D2R), respectively.

We further confirmed the colocalization of H3R with D1R and D2R in the striatum using specific antibodies in wildtype striatal slices, using antibodies that are widely used in the literature and have been validated in KO mice (53, 54). We applied similar analyses to confocal images and found colocalization of H3R with both D1R and D2R (Fig. 2, *D* and *E*). In this case, about 52% of D1R-positive and 59% D2R-postive cells coexpressed H3R. In the converse analysis, 45% of H3R-postive cells coexpressed D1R and 40% coexpressed D2R. The abundant expression of H3R in D1R- and D2R-SPNs is consistent with previous findings (26, 27).

### Modulation of signaling in D2R-SPNs by H3R-D2R coactivation

We examined key signaling pathways in SPNs to characterize the neural correlates of the interactive behavioral effects shown in Figure 1. We investigated changes of phosphorylation levels of several signaling molecules in D1R- and D2R-SPNs, using phospho-specific antibodies validated in our previous studies, and by many others (41, 55). We further validated the specificity of these phosphoantibodies by treating brain slices with calf intestinal alkaline phosphatase (CIAP) prior to immunostaining; CIAP-treated sections showed almost complete loss of immunofluorescence for each phospho-specific antibody, relative to buffer-treated sections stained in parallel with the same antibody (Fig. S2). CIAP treatment did not affect immunostaining using pan-Akt antibody (Fig. S2), suggesting it does not broadly affect the epitope availability or antibody binding.

We assessed the impact of DA and HA agonists on phosphorylation of several well-characterized signaling molecules (Akt, GSK3β, MSK1, and rpS6). Brain sections were processed in three batches; all values were normalized to the respective batch mean, to control for any batch effects. We used an automated object-based quantification approach, which identified D1R- and D2R-SPNs based on the FLAG and Myc epitopes, respectively. We then counted the proportion of D1R- and D2R-SPNs that also showed positive immunostaining for the target phosphoproteins and quantified staining density for each phosphoprotein within identified D1R- and D2R-SPNs.

**Figure 3:**
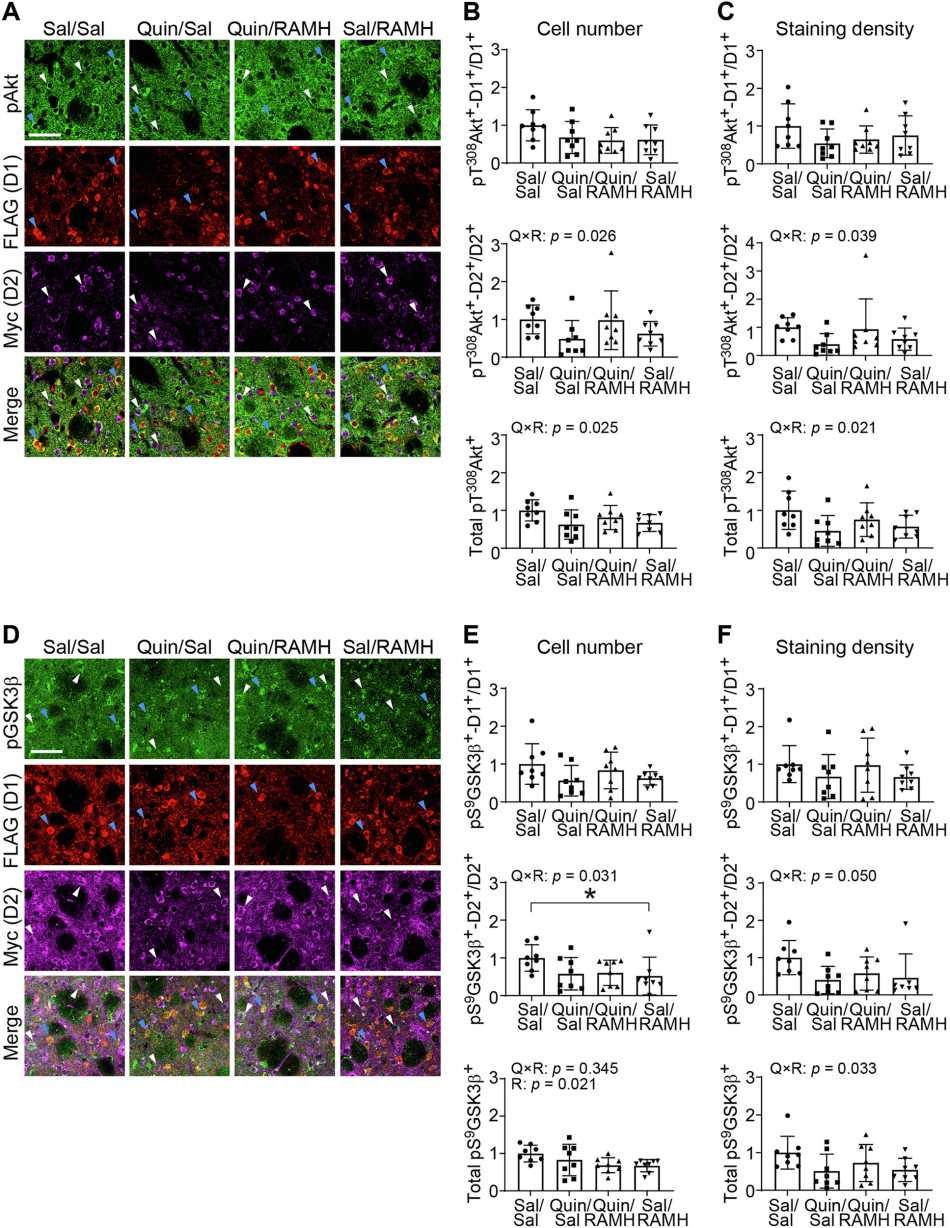
**Modulation of Akt–GSK3β signaling in D2R-SPNs by H3R and D2R coactivation.** Male and female D1-FLAG/D2-Myc mice received reserpine (2 mg/kg, s.c.) 20 h prior to drug administration. Mice received injections of saline (Sal) or RAMH (45 mg/kg, i.p.), followed by Sal or quinpirole (Quin, 0.5 mg/kg, i.p.) and anesthetized 30 min after treatment. *A*, representative images of pT^308^ Akt immunostaining in D1R- and D2R-SPNs. *Blue* and *white arrowheads* indicate D1R-SPNs (labeled by the FLAG epitope) and D2R-SPNs (labeled by the Myc epitope), respectively. Merged images were obtained by overlaying three channels from the same field of view. Scale bar represents 50 μm. *B*, the proportion of pT^308^ Akt-positive cells in D1R- and D2R-SPNs and the total number of pT^308^ Akt-positive cells. *Middle panel,* Quin × RAMH interaction: *F*(1, 28) = 5.569, *p* = 0.026, *η*^*2*^_*p*_ = 0.166. *Lower panel,* Quin × RAMH interaction: *F*(1, 28) = 5.638, *p* = 0.025, *η*^*2*^_*p*_ = 0.168. *C*, staining density of pT^308^ Akt-positive cells in D1R- and D2R-SPNs and the total fluorescence density of pT^308^ Akt-positive cells. *Middle panel,* Quin × RAMH: *F*(1, 28) = 4.681, *p* = 0.039, *η*^*2*^_*p*_ = 0.143. *Lower panel,* Quin × RAMH: *F*(1, 28) = 6.025, *p* = 0.021, *η*^*2*^_*p*_ = 0.177. *D*, representative images of pS^9^ GSK3β immunostaining in D1R- and D2R-SPNs. *Blue* and *white arrowheads* indicate D1R-SPNs (labeled by the FLAG epitope) and D2R-SPNs (labeled by the Myc epitope), respectively. Merged images were obtained by overlaying three channels from the same field of view. Scale bar represents 50 μm. *E*, the proportion of pS^9^ GSK3β-positive cells in D1R- and D2R-SPNs and the total number of pS^9^ GSK3β-positive cells. *Middle panel,* Quin × RAMH: *F*(1, 28) = 5.176, *p* = 0.0307, *η*^*2*^_*p*_ = 0.156. *Lower panel,* main effect of RAMH: *F*(1, 28) = 5.982, *p* = 0.021, *η*^*2*^_*p*_ = 0.176. *F*, staining density of pS^9^ GSK3β-positive cells in D1R- and D2R-SPNs and the total fluorescence density of pS^9^ GSK3β-positive cells. *Middle panel,* Quin × RAMH: *F*(1, 28) = 4.229, *p* = 0.050, *η*^*2*^_*p*_ = 0.135. *Lower panel,* Quin × RAMH: *F*(1, 28) = 5.049, *p* = 0.0327, *η*^*2*^_*p*_ = 0.153. All values are expressed as mean ± SD. Values were normalized to the corresponding Sal/Sal (each animal received two injections) group in each graph, respectively. Statistical analysis was performed using two-way ANOVAs in GraphPad Prism 9. See Table S2 for additional statistical details. Where significant drug interactions or main effects were found, multiple comparisons were conducted using post hoc Tukey test. ∗*p* < 0.05, n = 8 each group. Akt, serine/threonine PKB; D2R, dopamine 2 receptor; GSK3β, glycogen synthase kinase 3 beta; H3R, histamine H3 receptor; RAMH, R-(−)-α-methylhistamine dihydrobromide; SPN, spiny projection neuron.

As predicted, the D2/D3 agonist Quin altered signaling in D2R-SPNs but not in D1R-SPNs, in reserpinized mice (Fig. 3*A*). We first examined the Akt signaling upon D2R and H3R coactivation. There was a significant Quin × RAMH interaction in pT^308^ Akt-positive cells in all striatal cells (2 × 2 ANOVA: *p* = 0.025, *η*^*2*^_*p*_ = 0.168) and in D2R-SPNs (*p* = 0.026, *η*^*2*^_*p*_ = 0.166) but not in D1R-SPNs (Fig. 3*B*). Similarly, when we quantified pT^308^ Akt staining density, there was a significant Quin × RAMH interaction in all striatal cells (*p* = 0.021, *η*^*2*^_*p*_ = 0.177) and in D2R-SPNs (*p* = 0.039, *η*^*2*^_*p*_ = 0.143) but not in D1R-SPNs (Fig. 3*C*). Most pairwise post hoc comparisons did not reach statistical significance, but Quin treatment generally decreased phospho-Akt levels in D2R-SPNs, an effect that was reversed by RAMH cotreatment. RAMH alone led to nominal decreases in pAkt staining in D2R-SPNs, qualitatively consistent with our previous findings (41, 55).

There were similar interactive effects on phosphorylation of GSK3β, a key substrate of Akt (Fig. 3*D*). When we quantified pS^9^ GSK3β-positive cells, there was a significant Quin × RAMH interaction in D2R-SPNs (*p* = 0.031, *η*^*2*^_*p*_ = 0.156) but not in D1R-SPNs or in all cells (Fig. 3*E*). pS^9^GSK3β density showed a significant interaction in total cells (*p* = 0.033, *η*^*2*^_*p*_ = 0.153) and in D2R-SPNs (*p* = 0.050, *η*^*2*^_*p*_ = 0.135) but not in D1R-SPNs (Fig. 3*F*).

We next examined the downstream targets of MAPK signaling pathway in D1R- and D2R-SPNs after H3R and D2R coactivation. Previously, work has demonstrated H3R–D1R interactive effects on the MAPK in D1R-SPNs (27, 41). We found no Quin × RAMH interactions on the MAPK pathway, evaluated using pMSK1 (Fig. S3) and p-rpS6 (Fig. S4) immunostaining, in either D1R- or D2R-SPNs (Table S2). Together, these results indicate that H3R coactivation modulates D2R-mediated Akt–GSK3β signaling in D2R-SPNs (56, 57, 58), without influencing downstream targets of MAPK signaling, at least under these conditions.

### Interactive effects of H3R activation on SPN signaling following D1R and D2R coactivation with Apo

Having shown effects of H3R activation on D1R signaling in D1R-SPNs (41) and on D2R signaling in D2R-SPNs (Fig. 3), we next examined the functional consequences of H3R activation when D1R and D2R are coactivated, which is presumably generally the case *in vivo* when they are activated by DA. We used Apo, a well-characterized coagonist, to achieve pharmacological coactivation of D1R and D2R in intact mice. Apo induces locomotor activation and stereotypic behaviors in mice (59, 60); here, we administered Apo (or Sal) in combination with RAMH (or Sal) and examined the effects on behavior in the open field. These mice were not pretreated with reserpine and were not treated repeatedly; baseline activity did not differ among groups. Nevertheless, we kept baseline activity as a covariate in the model (two-way analysis of covariance) to match the analysis done in the Quin work. As in the Quin experiment, no effect of sex was found in a preliminary analysis, and so data from both sexes were combined in the primary analysis. There was a significant Apo × RAMH interaction in locomotor activity (distance traveled: *p* = 0.002, *η*^*2*^_*p*_ = 0.156; ambulatory activity: *p* = 0.002, *η*^*2*^_*p*_ = 0.125, Fig. S5, *A*–*C*). There were also significant Apo × RAMH interactions on stereotypic behaviors (stereotypic activity: *p* < 0.001, *η*^*2*^_*p*_ = 0.208; stereotypy time: *p* < 0.001, *η*^*2*^_*p*_ = 0.282, Fig. S5, *D* and *E*). Both Apo and RAMH enhanced stereotypy (Apo/Sal *versus* Veh/Sal, stereotypic activity: *p* = 0.002; stereotypy time: *p* < 0.001; Veh/Sal *versus* Veh/RAMH, stereotypic activity: *p* = 0.021; stereotypy time: *p* = 0.050). This increased stereotypy was again reversed by cotreatment with Apo and RAMH (Apo/Sal *versus* Apo/RAMH, stereotypic activity: *p* = 0.003; stereotypy time: *p* < 0.001; Apo/RAMH *versus* Veh/RAMH, stereotypic activity: *p* = 0.032; stereotypy time: *p* = 0.014).

**Figure 4:**
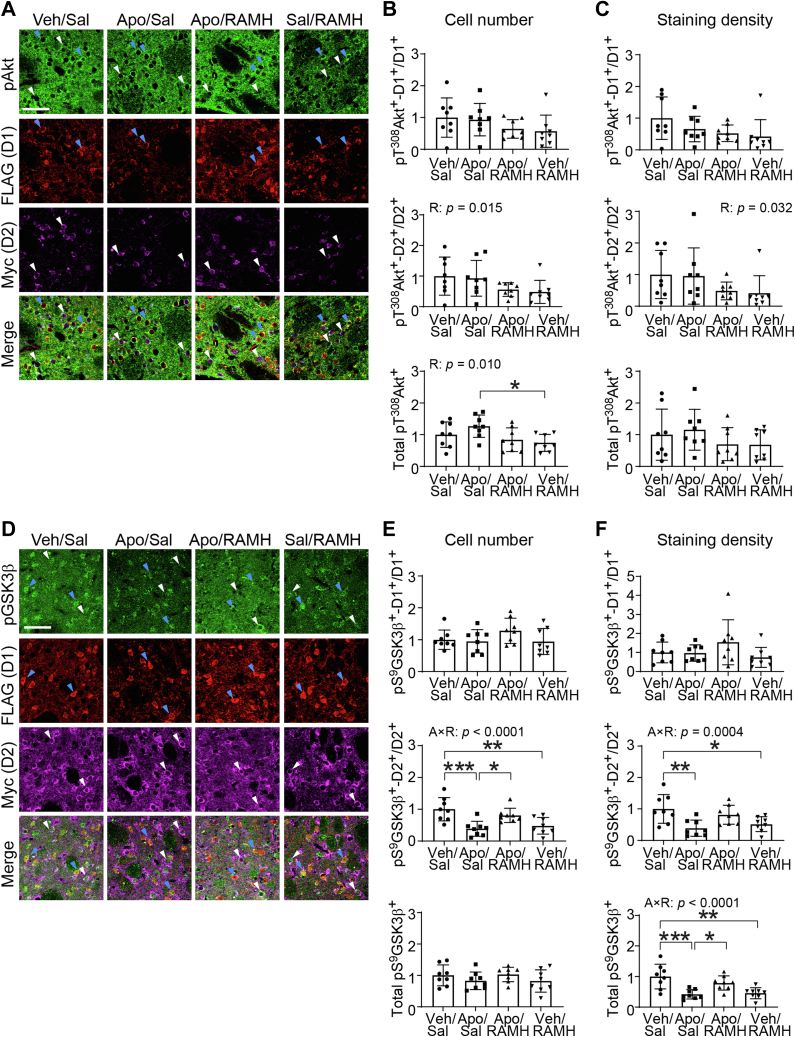
**Modulation of GSK3β signaling in D2R-SPNs by H3R–D2R interaction.** Male and female D1-FLAG/D2-Myc mice received injections of saline (Sal) or RAMH (45 mg/kg, i.p.), followed by vehicle (Veh) or apomorphine (Apo, 2 mg/kg, s.c.), and anesthetized 30 min after treatment. *A*, representative images of pT^308^ Akt immunostaining in D1R- and D2R-SPNs. *Blue* and *white arrowheads* indicate D1R-SPNs (labeled by the FLAG epitope) and D2R-SPNs (labeled by the Myc epitope), respectively. Merged images were obtained by overlaying three channels from the same field of view. Scale bar represents 50 μm. *B*, the proportion of pT^308^ Akt-positive cells in D1R- and D2R-SPNs and the total number of pT^308^ Akt-positive cells. *Middle panel,* main effect of RAMH: *p* = 0.015, *η*^*2*^_*p*_ = 0.195. *Lower panel,* RAMH: *p* = 0.010, *η*^*2*^_*p*_ = 0.214. *C*, staining density of pT^308^ Akt-positive cells in D1R- and D2R-SPNs and the total fluorescence density of pT^308^ Akt-positive cells. *Middle panel,* RAMH: *p* = 0.032, *η*^*2*^_*p*_ = 0.154. *D*, representative images of pS^9^ GSK3β immunostaining in D1R- and D2R-SPNs. *Blue* and *white arrowheads* indicate D1R-SPNs (labeled by the FLAG epitope) and D2R-SPNs (labeled by the Myc epitope), respectively. Merged images were obtained by overlaying three channels from the same field of view. Scale bar represents 50 μm. *E*, the proportion of pS^9^ GSK3β-positive cells in D1R- and D2R-SPNs and the total number of pS^9^ GSK3β-positive cells. *Middle panel,* Apo × RAMH: *p* < 0.0001, *η*^*2*^_*p*_ = 0.450. *F*, staining density of pS^9^ GSK3β-positive cells in D1R- and D2R-SPNs and the total fluorescence density of pS^9^ GSK3β-positive cells. *Middle panel,* Apo × RAMH: *p* = 0.0004, *η*^*2*^_*p*_ = 0.362. *Lower panel,* Apo × RAMH: *p* < 0.0001, *η*^*2*^_*p*_ = 0.469. All values are expressed as mean ± SD. Values were normalized to the corresponding Veh/Sal group in each graph. Statistical analysis was performed using two-way ANOVAs in GraphPad Prism 9. See Table S2 for additional statistical details. Where significant drug interactions or main effects were found, multiple comparisons were conducted using post hoc Tukey test. ∗*p* < 0.05, ∗∗*p* < 0.01, ∗∗∗*p* < 0.001, n = 8 each group. D1R, dopamine 1 receptor; D2R, dopamine 2 receptor; GSK3β, glycogen synthase kinase 3 beta; H3R, histamine H3 receptor; SPN, spiny projection neuron; RAMH, R-(−)-α-methylhistamine dihydrobromide.

Having confirmed functional interactions of H3R and D1R/D2R on behavior, we investigated changes in signaling in D1R- and D2R-SPNs. To our surprise, in the case of Akt, there were no significant Apo × RAMH interaction on pT^308^ Akt levels in either D1R- or D2R-SPNs; there was a main effect of RAMH in D2R-SPNs (Fig. 4, *A–C*). GSK3β, in contrast, showed Apo × RAMH interactive effects in D2 cells consistent with those seen after Quin. There was a significant Apo × RAMH interaction in the number of pS^9^ GSK3β D2R-SPNs (*p* < 0.0001, *η*^*2*^_*p*_ = 0.450) but not in D1R-SPNs or total cells (Fig. 4*E*). Similarly, there was an Apo × RAMH interaction in pS^9^ GSK3β staining density in total cells (*p* < 0.0001, *η*^*2*^_*p*_ = 0.469) and D2R-MSNs (*p* = 0.0004, *η*^*2*^_*p*_ = 0.362) but not in D1R-MSNs (Fig. 4*F*). Pairwise post hoc comparisons revealed that both Apo and RAMH decreased pS^9^ GSK3β in D2R-SPNs, but these effects were reversed by Apo–RAMH coadministration (Fig. 4, *E* and *F*). The effect of RAMH on pS^9^ GSK3β in D2R-SPNs is consistent with our previous work (41, 55).

We expected that activation of D1R on D1R-SPNs would have effects on the MAPK signaling pathway, measured by phosphorylation of MSK1 and rpS6, similar to those seen with the D1R agonist SKF82958 in our previous work (41). However, we did not observe main effects of Apo, or Apo × RAMH interactive effects, on phosphorylation of MSK1 (Fig. S6). There were significant main effects of RAMH on the number of pT^581^ MSK1/D1R cells (*p* = 0.0210, *η*^*2*^_*p*_ = 0.176), pT^581^ MSK1/D2R cells (*p* = 0.008, *η*^*2*^_*p*_ = 0.225), and total pT^581^ MSK1-positive cells (*p* = 0.037, *η*^*2*^_*p*_ = 0.146; Fig. S6*B*), consistent with the RAMH effects in our previous study (41). No drug effects were found in staining density of pT^581^ MSK1 in D1R- or D2R-SPNs.

There were significant Apo × RAMH interactions on the phosphorylation of rpS6 at S^235/236^ in all cells (*p* = 0.021, *η*^*2*^_*p*_ = 0.176) and in D1R-SPNs (*p* = 0.0497, *η*^*2*^_*p*_ = 0.131) but not in D2R-SPNs (Fig. S7). However, post hoc examination revealed that Apo decreases pS^235/236^ rpS6 levels, which contrasts with our previous observations with SKF82958 (41). A main effect of RAMH was found on the number of pS^235/236^ rpS6 D1R-SPNs (*p* = 0.0120, *η*^*2*^_*p*_ = 0.205). There were no changes in pS^235/236^ rpS6 in D2 cells.

Regarding pS^240/244^ rpS6 (Fig. S7, *D*–*F*), main effects of Apo were observed on the number of total pS^240/244^ rpS6 cells (*p* = 0.037, *η*^*2*^_*p*_ = 0.146) and of pS^240/244^ rpS6-positive D1R-SPNs (*p* = 0.038, *η*^*2*^_*p*_ = 0.144). A main effect of RAMH on pS^240/244^ rpS6 staining density was found in D1R-SPNs (*p* = 0.016, *η*^*2*^_*p*_ = 0.191). There were no changes of pS^240/244^ rpS6 in D2 cells. In total, the effects of the dual agonist Apo were mixed and did not wholly replicate effects on MAPK signaling reported previously in D1R-SPNs with the D1R-specific agonist SKF82958 (41). In D2R-SPNs, however, Apo and RAMH showed a robust interaction on pGSK3β signaling in D2R-SPNs, in agreement with our predictions and with the results seen with Quin in reserpinized mice (Fig. 3).

As a robustness check, we analyzed pAkt and pGSK3β staining with an intensity-based approach using Manders’ colocalization coefficient as a measure of the degree of pixel overlap/colocalization between markers (61) (Fig. S8). The results of this reanalysis of pGSK3β were consistent with those obtained using the object-based approach (Figs. 3 and 4). Using colocalization coefficients, a trend-level Quin × RAMH interaction (*p* = 0.083) and a significant Apo × RAMH interaction (*p* = 0.003, *η*^*2*^_*p*_ = 0.268) were found in D2R-SPNs. Using total density of colocalized voxels, both interactions were significant in D2R-SPNs (Quin × RAMH: *p* = 0.038, *η*^*2*^_*p*_ = 0.144; Apo × RAMH: *p* = 0.007, *η*^*2*^_*p*_ = 0.231). There were no significant interactions in D1R-SPNs and no consistent effects in pAkt staining.

In summary, we found consistent and robust interactive effects on the modulation of GSK3β signaling by H3R and D2R agonists in D2R-SPNs, using different agonists and analytic approaches.

### H3R agonist treatment modulates the effects of D2R activation on Akt–GSKβ signaling in striatal lysates

**Figure 5:**
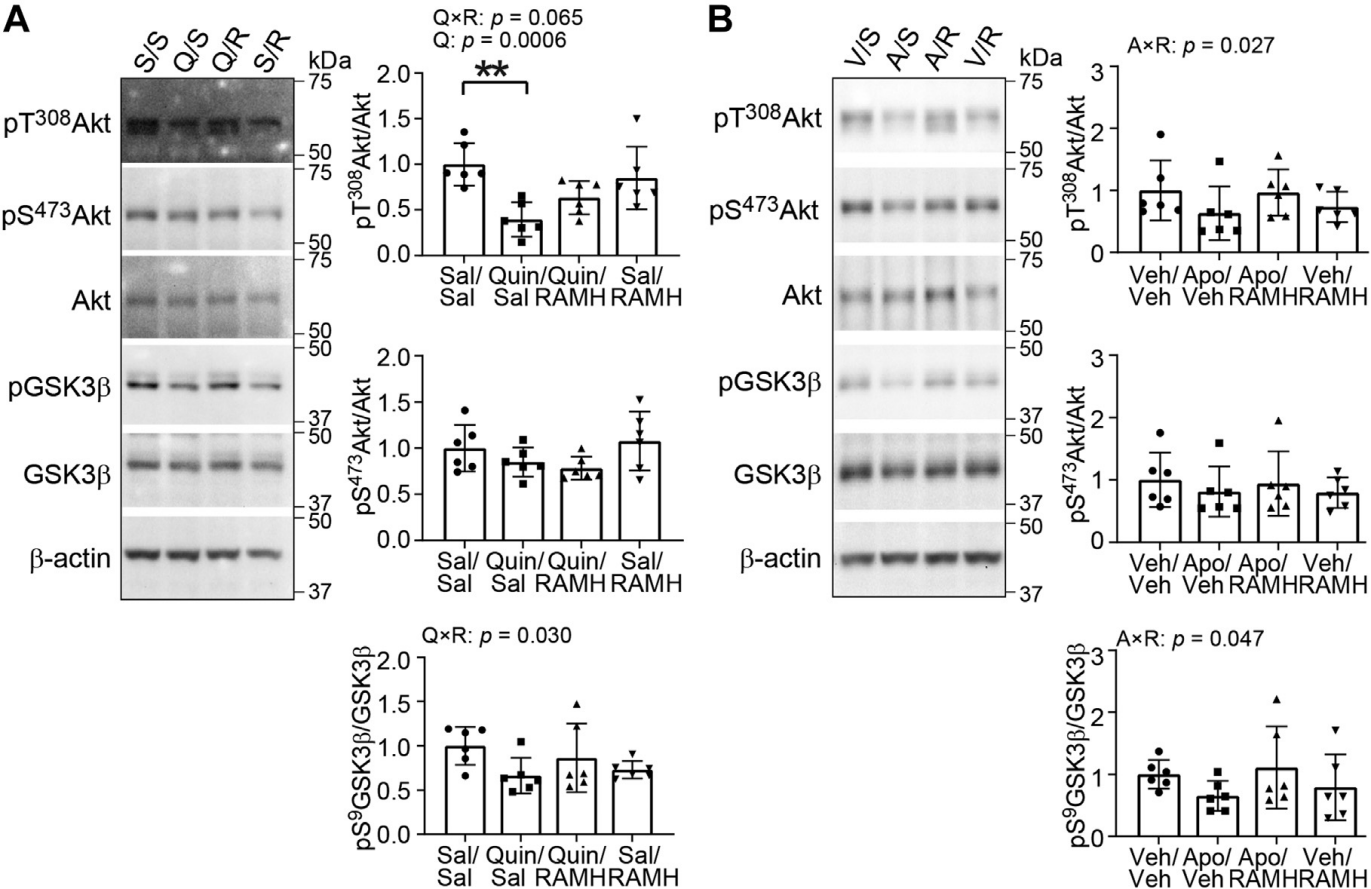
**H3R agonist treatment modulates the effects of D2R activation on Akt–GSKβ signaling in mouse striatal lysates.** Male and female C57BL/6J mice received reserpine (2 mg/kg, s.c.) 20 h prior to drug administration. Mice received injections of saline (Sal) or RAMH (45 mg/kg, i.p.), followed by Sal or quinpirole (Quin, 0.5 mg/kg, i.p.), and sacrificed 30 min after treatment (*A*). Naïve C57BL/6J mice received injections of Sal or RAMH (45 mg/kg, i.p.), followed by vehicle (Veh) or apomorphine (Apo, 2 mg/kg, s.c.), and sacrificed 30 min after treatment (*B*). After treatment, phosphoprotein and total protein levels were assayed using Western blotting. Phosphorylation levels of Akt and GSK3β were normalized to corresponding total protein levels. Pan-protein levels were normalized to β-actin as a loading control. *A*, phosphorylation and total protein levels of signal molecules in the striatal lysates after Quin–RAMH treatment. pT^308^ Akt, Quin × RAMH interaction: *F*(1, 20) = 3.810, *p* = 0.065; main effect of Quin: *F*(1, 20) = 16.7, *p* = 0.0006, *η*^*2*^_*p*_ = 0.316. pS^9^ GSK3β, Quin × RAMH interaction: *F*(1, 20) = 5.450, *p* = 0.030, *η*^*2*^_*p*_ = 0.214. *B*, phosphorylation and total protein levels of signal molecules in the striatal lysates after Apo–RAMH treatment. pT^308^ Akt, Apo × RAMH interaction: *F*(1, 20) = 5.660, *p* = 0.027, *η*^*2*^_*p*_ = 0.221. pS^9^ GSK3β, Apo × RAMH interaction: *F*(1, 20) = 4.500, *p* = 0.047, *η*^*2*^_*p*_ = 0.184. All values are expressed as mean ± SD. Values were normalized to the corresponding Sal/Sal in A (or Veh/Sal in B) group in each graph. Statistical analysis was performed using two-way ANOVAs (raw values for *A*; aligned rank transformed values for *B*) in GraphPad Prism 9. See Table S2 for additional statistical details. Where significant drug interactions or main effects were found, multiple comparisons were conducted using post hoc Tukey test. ∗∗*p* < 0.01, n = 6 each group. Representative images in *A* were reproduced from highlighted lanes in Fig. S11*A* (Fig. S11, *A* and *B* shows the entire dataset for this experiment). Representative images in *B* were reproduced from highlighted lanes in Fig. S11*D* (Fig. S11, *C* and *D* shows the entire dataset for this experiment). Akt, serine/threonine PKB; D2R, dopamine 2 receptor; H3R, histamine H3 receptor; GSKβ, glycogen synthase kinase 3 beta; RAMH, R-(−)-α-methylhistamine dihydrobromide.

We next tested the interaction of D2R and H3R activation on Akt–GSKβ signaling in striatal lysates; these experiments complement the aforementioned analyses using immunohistochemistry (Figs. 3, *C* and *F* and 4, *C* and *F*). Mice were treated with Sal or Quin in reserpinized mice or with vehicle or Apo in intact mice, followed by Sal or RAMH injections; their striata were rapidly dissected, and protein was extracted in the presence of phosphatase inhibitors. Phosphoprotein and total protein levels of Akt and GSK3β were probed using Western blotting (WB). We found Quin × RAMH and Apo × RAMH interactions on Akt phosphorylation at T^308^, which is modulated by D2R–β-arrestin 2 signaling (56, 57) (Quin × RAMH: *p* = 0.065, Fig. 5*A*; Apo × RAMH: *p* = 0.027, *η*^*2*^_*p*_ = 0.221, Fig. 5*B*) but not on phosphorylation at S^473^. Similarly, we found significant Quin × RAMH and Apo × RAMH interactions on pGSK3β (Quin × RAMH: *p* = 0.030, *η*^*2*^_*p*_ = 0.214, Fig. 5*A*, *lower panel*; Apo × RAMH: *p* = 0.047, *η*^*2*^_*p*_ = 0.184, Fig. 5*B*, *lower panel*). Again, total Akt and GSK3β levels were not changed by Quin/RAMH or Apo/RAMH treatments (Figs. S9 and S11). Together, these results confirmed functional consequences of H3R–D2R coactivation on the modulation of pT^308^ Akt and pS^9^ GSK3β, in agreement with the immunostaining data (Figs. 3 and 4).

### H3R agonist treatment modulates the β-arrestin 2–PP2A–Akt signaling complex in response to D2R activation in the mouse striatum

The β-arrestin 2–PP2A–Akt signaling complex mediates G protein–independent signaling downstream of D2R activation (56). We investigated this signaling complex after Quin or Apo treatment and the effects of H3R activation. Akt was immunoprecipitated from striatal lysates (used in WB as described previously) using an antibody used previously for this purpose (56). We first confirmed that Akt-containing immune complex was specifically pulled down by the anti-Akt antibody but not the isotype control immunoglobulin G (IgG) (not shown). Binding partners including β-arrestin 2, PP2A regulatory subunit (B-sub), and PP2A catalytic subunit (C-sub) were probed using WB; their levels were normalized to immunoprecipitated Akt levels.

**Figure 6:**
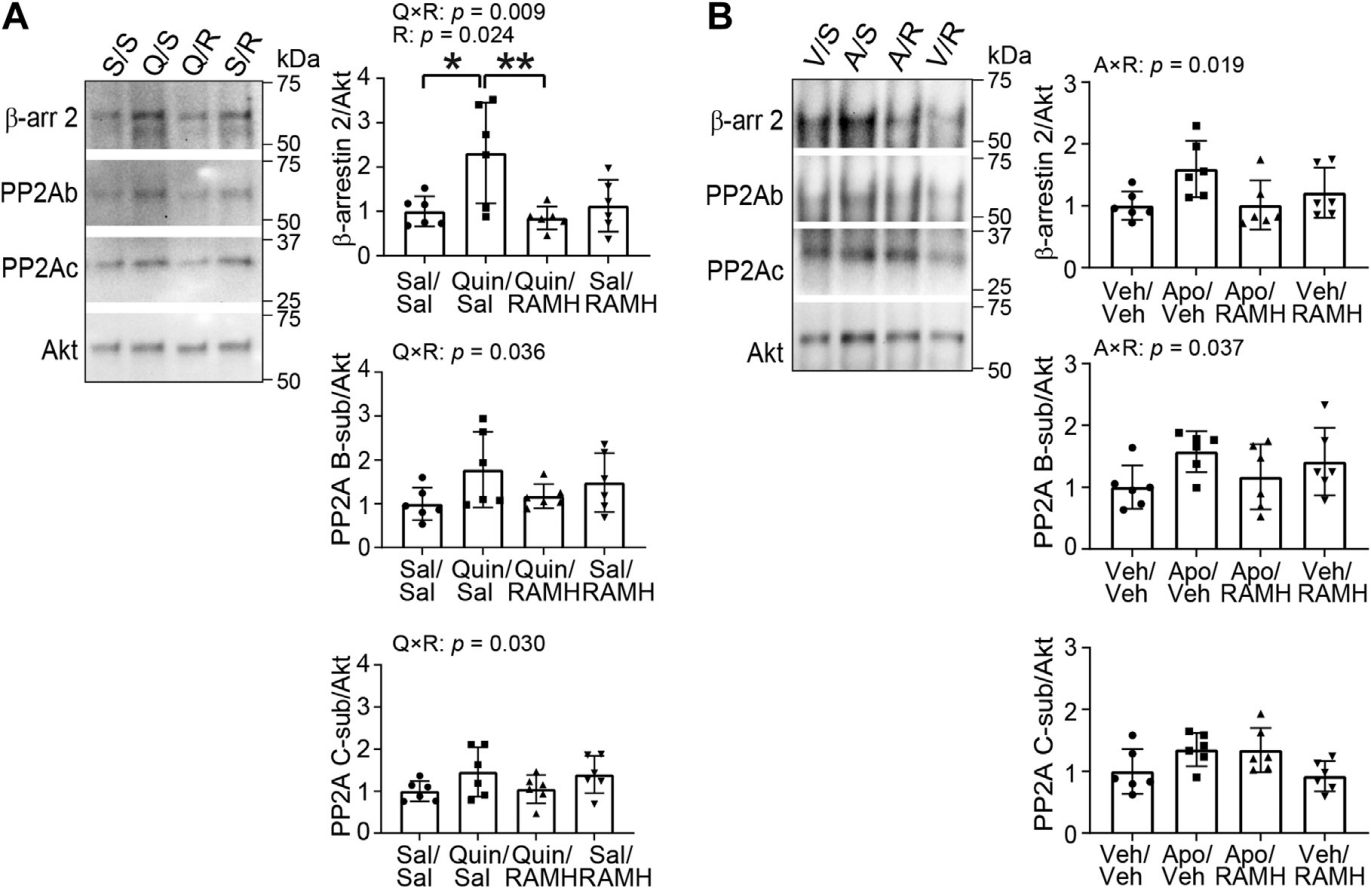
**H3R agonist treatment modulates the β-arrestin 2–PP2A–Akt signaling complex in response to D2R activation in the mouse striatum.** Male and female C57BL/6J mice received reserpine (2 mg/kg, s.c.) 20 h prior to drug administration. Mice received injections of saline (Sal) or RAMH (45 mg/kg, i.p.), followed by Sal or quinpirole (Quin, 0.5 mg/kg, i.p.), and were sacrificed 30 min after treatment (*A*). Naïve C57BL/6J mice received injections of Sal or RAMH (45 mg/kg, i.p.), followed by vehicle (Veh) or apomorphine (Apo, 2 mg/kg, s.c.) and sacrificed 30 min after treatment (*B*). After treatment, Akt was immunoprecipitated from striatal lysates using anti-Akt antibody conjugated to Sepharose bead. Coimmunoprecipitation of β-arrestin 2 and PP2A subunits was normalized to immunoprecipitated Akt levels. *A*, association of β-arrestin 2 and PP2A subunits with Akt after Quin–RAMH treatment. β-arrestin 2, Quin × RAMH interaction: *F*(1, 20) = 8.410, *p* = 0.009, *η*^*2*^_*p*_ = 0.230; main effect of RAMH: *F*(1, 20) = 5.980, *p* = 0.024, *η*^*2*^_*p*_ = 0.296. PP2A B-subunit, Quin × RAMH: *F*(1, 20) = 5.070, *p* = 0.036, *η*^*2*^_*p*_ = 0.202. PP2A C-subunit, Quin × RAMH: *F*(1, 20) = 5.460, *p* = 0.030, *η*^*2*^_*p*_ = 0.215. *B*, association of β-arrestin 2 and PP2A subunits with Akt after Apo–RAMH treatment. β-arrestin 2, Apo × RAMH interaction: *F*(1, 20) = 6.470, *p* = 0.0193, *η*^*2*^_*p*_ = 0.245. PP2A B-subunit, Apo × RAMH: *F*(1, 20) = 4.970, *p* = 0.0374, *η*^*2*^_*p*_ = 0.199. PP2A B-subunit, main effect of Apo: *F*(1, 20) = 9.260, *p* = 0.006, *η*^*2*^_*p*_ = 0.316. All values are expressed as mean ± SD. Values were normalized to the corresponding Sal/Sal in *A* or Veh/Sal in *B* (each animal received two injections) group in each graph, respectively. Statistical analysis was performed using two-way ANOVAs in GraphPad Prism 9. See Table S2 for additional statistical details. Where significant drug interactions or main effects were found, multiple comparisons were conducted using post hoc Tukey test. ∗*p* < 0.05, ∗∗*p* < 0.01, n = 6 each group. Representative images in *A* were reproduced from highlighted lanes in Fig. S12*B* (Fig. S12, *A* and *B* shows the entire dataset for this experiment). Representative images in *B* were reproduced from highlighted lanes in Fig. S12*C* (Fig. S12, *C* and *D* shows the entire dataset for this experiment). Akt, serine/threonine PKB; H3R, histamine H3 receptor; D2R, dopamine 2 receptor; RAMH, R-(−)-α-methylhistamine dihydrobromide.

We found significant Quin × RAMH interactions on the composition of the Akt signaling complex (β-arrestin 2: *p* = 0.009, *η*^*2*^_*p*_ = 0.230; PP2A B-sub: *p* = 0.0358, *η*^*2*^_*p*_ = 0.202; PP2A C-sub: *p* = 0.030, *η*^*2*^_*p*_ = 0.215; Figs. 6*A* and S12, *A* and *B*), and similarly significant Apo × RAMH interactions (β-arrestin 2: *p* = 0.019, *η*^*2*^_*p*_ = 0.245; PP2A B-sub: *p* = 0.037, *η*^*2*^_*p*_ = 0.199, Figs. 6*B* and S12, *C* and *D*). Quin treatment increased the association of β-arrestin 2 with Akt (*p* = 0.014), and H3R coactivation blocked this effect (Quin/RAMH *versus* Quin/Sal: *p* = 0.014, Fig. 6*A*, *upper panel*). Total protein levels of these molecules were unchanged in the lysates prior to Akt immunoprecipitation (Figs. S10 and S11).

These findings suggest that the interactive effects of H3R and D2R agonists on the phosphorylation of Akt and GSK3β seen in Figure 5 are likely to be mediated by β-arrestin 2, consistent with previous studies (56, 57). To investigate whether Akt and GSK3β could be modulated through convergence of G protein–dependent signaling in response to H3R and D2R coactivation, we examined several well-known PKA substrates at the sites that are modulated by PKA (pS^845^ of GluA1 (glutamate ionotropic receptor AMPA type subunit 1), pS^133^ of cAMP response element–binding protein , and pT^34^ of DARPP-32) as markers of cAMP–PKA signaling (62, 63, 64) in the same samples described in Figure 5. We found significant main effects of Quin treatment on the phosphorylation of all three PKA substrates in reserpinized mice (pS^845^ GluA1: *p* = 0.017, *η*^*2*^_*p*_ = 0.252; pS^133^ cAMP response element–binding protein: *p* = 0.042, *η*^*2*^_*p*_ = 0.190; pT^34^ DARPP-32: *p* = 0.028, *η*^*2*^_*p*_ = 0.218), consistent with the regulation of cAMP–PKA signaling by the Gαi/o-coupled D2R upon agonist treatment (reviewed in Ref. (65)). No Quin × RAMH interactions were observed in the phosphorylation levels of these targets (Figs. S13*A* and S14*A*), suggesting coactivation of H3R did not antagonize D2R-induced cAMP–PKA signaling. Consistent with our previous study showing that acute RAMH treatment did not alter cAMP–PKA signaling, at least under the conditions examined (41). Acute drug treatment did not alter total protein levels (Figs. S13*A* and S14*A*).

We also examined PKA-mediated phosphorylation of these molecules upon acute Apo treatment. In this case, no statistically significant effects of Apo, RAMH, or Apo × RAMH interactions were found, although Apo showed some effects on pS^845^ GluA1 (*p* = 0.163), and pT^34^ DARPP-32 (*p* = 0.126) at trend level. Total protein levels remained unchanged (Figs. S13*B* and S14*B*). To confirm the lack of interactive effects of H3R and D2R coactivation on the phosphorylation of PKA substrates was not because of technical issues, we reprobed Akt and GSK3β on the same sets of blots. We replicated the findings in Figure 5 and found drug interactions on the levels of pT^308^ Akt (at trend level) and pS^9^ GSK3β but not pS^473^ Akt in Quin and RAMH-treated samples (Figs. S15*A* and S16*A*) as well as in Apo and RAMH-treated samples (Figs. S15*B* and S16*B*). Together, these results suggest that G protein–dependent cAMP–PKA signaling is unlikely to play a major role in the modulation of Akt/GSK3β signaling in D2-SPNs upon H3R and D2R coactivation (Figure 3, Figure 4, Figure 5).

### Coprecipitation and close proximity of H3R and D2R in the mouse striatum

**Figure 7:**
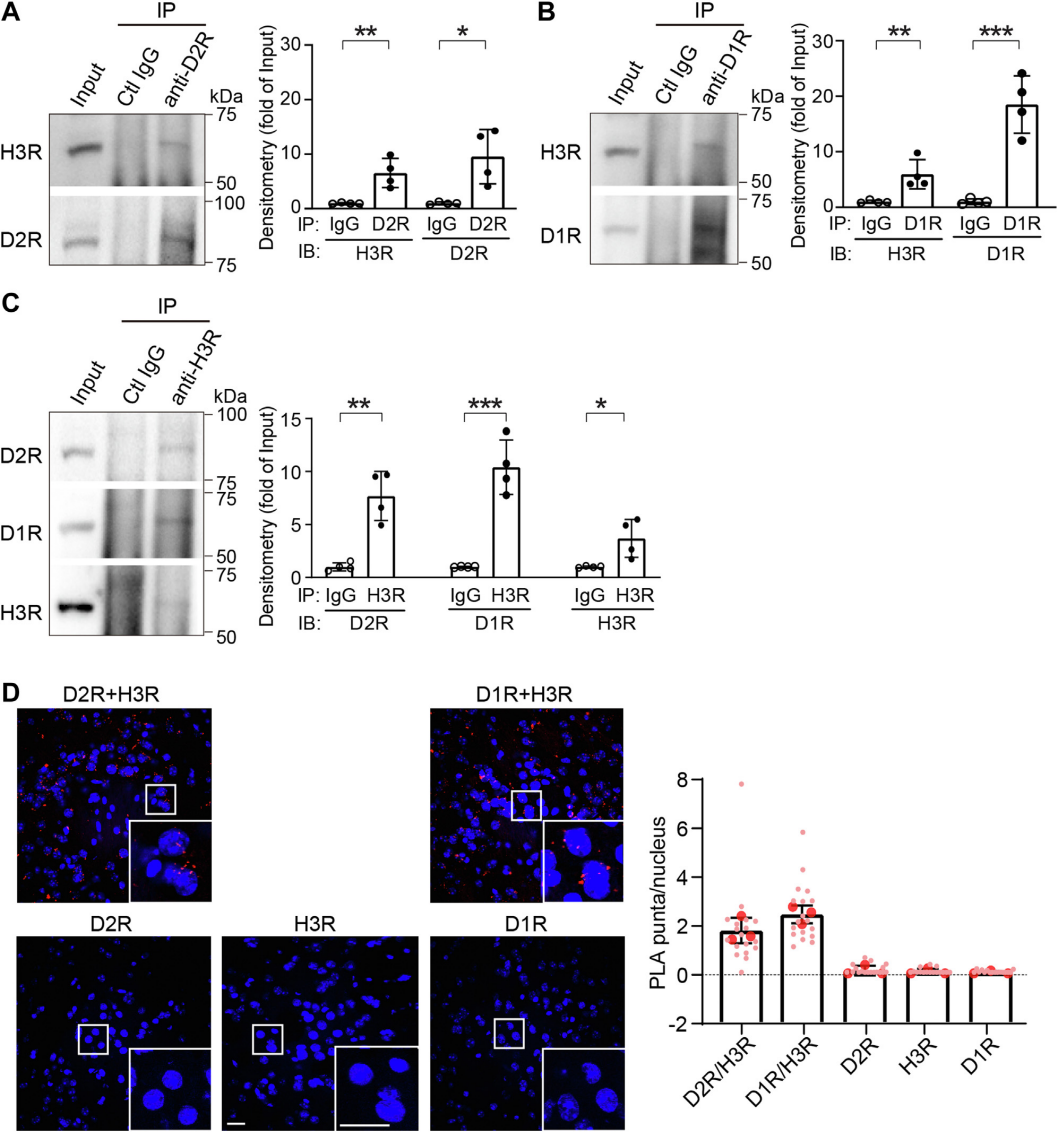
**Interaction and proximity of H3R and D2R in the mouse striatum.** Striatal lysates from naïve male and female C57BL/6J mice were used for immunoprecipitation with anti-D2R antibody (*A*), anti-D1R antibody (*B*), or anti-H3R antibody (*C*). Isotype immunoglobulins (IgGs) were used as negative controls. Coimmunoprecipitated binding partners were assayed on Western blotting. *A*, coimmunoprecipitation of H3R by anti-D2R antibody. H3R: *t*(6) = 4.12, *p* = 0.006, *d* = 2.910; D2R: *t*(6) = 3.430, *p* = 0.014, *d* = 2.424. *B*, coimmunoprecipitation of H3R by anti-D1R antibody. H3R: *t*(6) = 3.730, *p* = 0.010, *d* = 2.635; D1R: *t*(6) = 6.740, *p* < 0.001, *d* = 4.767. *C*, coimmunoprecipitation of D2R and D1R by anti-H3R antibody. D2R: *t*(6) = 5.700, *p* = 0.001, *d* = 4.027; D1R: *t*(6) = 7.330, *p* < 0.001, *d* = 5.181; H3R: *t*(6) = 2.990, *p* = 0.024, *d* = 2.118. All values are expressed as mean ± SD. Immunoprecipitated proteins were normalized to their levels in the corresponding inputs in each graph, respectively. Statistical analysis was performed using unpaired two-tailed *t* tests in GraphPad Prism 9. ∗*p* < 0.05, ∗∗*p* < 0.01, ∗∗∗*p* < 0.001, n = 4 each group. *D*, localization of H3R in close proximity to D2R or D1R was accessed using proximity ligation assay (PLA). Striatal sections from naïve male and female C57BL/6J mice were immunostained using D2R–H3R antibody pair and several control groups. The D1R–H3R antibody pair was used as a positive control, whereas single antibody staining was performed as negative controls (see Fig. S18 for additional control groups). Positive PLA signal (when antibody pair labeled proteins in close proximity) was visualized as *red puncta* around cell nuclei counterstained by DAPI (*blue*). Scale bar represents 20 μm. Average number of *red puncta* per nucleus was used as a measure of PLA signal. All values are expressed as mean ± SD. n = 3 each group. DAPI, 4′,6-diamidino-2-phenylindole; D1R, dopamine 1 receptor; D2R, dopamine 2 receptor; H3R, histamine H3 receptor.

H3R coimmunoprecipitates with D1R and D2R in rat striatal lysates, suggesting that the receptors associate in a complex (27). We confirmed these findings in mouse striatal lysates using the same technique. D2R- and D1R-containing immune complexes were immunoprecipitated with specific anti-D2R (Fig. 7*A*) and anti-D1R (Fig. 7*B*) antibodies, respectively. Isotype IgG was used as a negative control. Coimmunoprecipitated H3R in these receptor complexes was visualized using WB. The molecular weight of these targets on WB were consistent with that previously described using the same antibodies (66, 67, 68). Ten percent of the amount of lysates used in immunoprecipitation was loaded on the WB to measure input protein concentration; immunoprecipitated proteins were normalized to their levels in this input. We observed pull-down of D2R and D1R by the corresponding antibodies but not by control antibodies, as expected (D2R: *p* = 0.014, *d* = 2.424; D1R: *p* < 0.001, *d* = 4.767, Fig. 7, *A* and *B*, last 2 bars). H3R was found to be coimmunoprecipitated by both anti-D2R (*p* = 0.006, *d* = 2.910, Fig. 7*A*, first 2 bars) and anti-D1R antibody (*p* = 0.010, *d* = 2.635, Fig. 7*B*, first 2 bars; Fig. S17) but not by IgG controls. We also did a complimentary experiment by immunoprecipitating H3R and measuring coprecipitated D2R and D1R. As predicted, we found copurification of both D2R and D1R with H3R in mouse striatal lysates (D2R: *p* = 0.001, *d* = 4.027; D1R: *p* < 0.001, *d* = 5.181, Figs. 7*C* and S17).

The PLA is widely used to identify proteins that are localized in close proximity to one another (69, 70). Positive PLA signal has been observed using specific antibodies against H3R and D1R (42, 71) but not previously for H3R and D2R. We tested H3R–D2R colocalization using PLA, with H3R–D1R as a positive control and single antibody staining as negative controls. Positive PLA signal *(red spots*) was observed when receptors were labeled with anti-H3R and anti-D2R or anti-D1R but not with only one of the antibodies (Fig. 7*D*). This indicates that H3R and D2R are localized in close proximity in mouse striatal sections, consistent with the formation of a receptor complex. We also carried out PLA with additional controls using antibodies against DA receptors and sigma receptors. We observed positive PLA signal with D1R–σ1R, D1R–σ2R, and D2R–σ1R antibody pairs but not D2R–σ2R antibody pair (Fig. S18), consistent with the findings in a previous report (72).

## Discussion

The striatum is the primary input nucleus of the basal ganglia circuitry. It sends projections *via* two distinct pathways, the direct (striatonigral) pathway, in which neurons preferentially express D1R, and the indirect (striatopallidal) pathway, in which neurons preferentially express D2R (2, 25, 73). These projections are differentially modulated by DA; the balance between them is crucial in modulating basal ganglia function and sculpting behavioral outputs (74, 75). There are extensive studies on the regulation of D1R- and D2R-SPNs by different neurotransmitters, including glutamate, DA, GABA, and acetylcholine (73, 76, 77). However, modulation of striatum-basal ganglia circuitry by HA is relatively poorly understood.

Recent findings on interactions between H3R and D1R or D2R in the striatum reinforce the functional importance of these putative receptor complexes in the brain. At the behavioral level, H3R–D1R interaction mitigates D1R agonist–induced locomotion (21, 41). A similar effect of H3R–D2R interaction on locomotion has also been described (21). At the cellular level, differential regulation of MAPK and Akt–GSKβ signaling in distinct cell types has been shown (41, 55). At the molecular level, copurification of H3R with D1R and D2R has been demonstrated in striatal lysates. Moreover, positive fluorescence signal has been detected in H3R–D1R or H3R–D2R cotransfected cells using bioluminescence resonance energy transfer (21), consistent with the idea of receptor heteromerization. Definitive evidence for direct and specific physical interactions between these receptors *in vivo* is still lacking.

In this work, we focused on the functional consequences of H3R–D2R interactions on behavior and cellular signaling *in vivo*. We confirmed previous findings that coactivation of H3R and D2R by selective agonists attenuates D2R agonist–induced locomotor activity. We also found interactive effects of H3R–D2R activation on stereotypy. We replicated previous findings that H3R is abundantly expressed in both D1R-SPNs and D2R-SPNs. We further showed that H3R and D2R are present in close physical proximity in the striatum, using PLA in mouse striatal brain sections. These results in intact brain are in agreement with previous bioluminescence resonance energy transfer findings in transfected cells (21).

Our most robust and interesting finding is the interactive modulation of Akt–GSK3β signaling by H3R and D2R in D2R-SPNs. We have shown regulation of Akt and GSK3β by H3R agonists in a previous study (41). Here, we extended these findings by examining the interactive effects of H3R and D2R activity on this signaling pathway. We observe significant interactions of H3R and D2R agonists on the regulation of GSK3β, with large effect sizes across two different D2R agonists and several technical approaches. Analogous to the pattern seen in D1R-SPNs (on MAPK regulation), coactivation of H3R and D2R attenuated the effects of D2R agonists alone. The interactive effects are more clearly seen in pGSK3β levels than in pAkt levels in Figure 3. GSK3β is a well-established downstream target of Akt. However, phosphorylation of GSK3β at Ser^9^ is also modulated by other kinases and phosphatase, such as PKA (78), PKC (79), PP1, and PP2A (80). Interestingly, GSK3β is also a component of the β-arrestin 2–PP2A–Akt complex (56, 81). We speculate that upon D2R activation and recruitment of the complex, GSK3β will be dephosphorylated by PP2A and less phosphorylated by Akt (which is dephosphorylated and inhibited by PP2A), resulting in greater changes in pGSK3β levels that can be better detected by immunostaining. This may, at least partially, contribute to the inconsistency of the interactive effects of H3R and D2R agonists on pAkt and pGSK3β.

The molecular mechanisms underlying these interactive effects may be, at least in part, through the β-arrestin 2 pathway, whose role in D2R signaling has been well established (56, 82). We replicated the modulation of the β-arrestin 2–PP2A–Akt signaling complex in response to D2R activation. Importantly, we found that coactivation of H3R disrupted the effects of D2R on this signaling complex (Fig. 6). It may be that H3R ligand binding to the H3R–D2R heterodimer allosterically disrupts the binding of agonist to the D2R receptor: a previous ligand-binding assay in striatal lysates showed that activation of H3R significantly decreases agonist binding to D2R (21). Other mechanisms may also contribute to the modulation of D2R signaling by H3R. Phosphorylation of D2R by GPCR kinases (GRKs, particularly GRK2 and GRK3) and PKC have been shown to facilitate recruitment of β-arrestin to D2R and initiate G protein–independent signaling or internalization of the receptor (83, 84, 85, 86, 87, 88). Activity or membrane targeting of GRKs is modulated by several kinases including PKC (89, 90), ERK1/2 (91), and PKA (92). Interestingly, activation of H3R has been shown to regulate PKC (93, 94), ERK1/2 (27, 94, 95), and PKA pathways (96), raising the possibility that H3R could modulate D2R–β-arrestin 2–dependent Akt/GSK3β signaling *via* crosstalk of these downstream pathways, rather than by direct modulation within receptor heteromers.

One limitation of the current work is that we did not definitively rule out the potential involvement of G protein–dependent mechanism in the action of β-arrestin 2, that is, whether it acts as an independent transducer or a supporter of G protein–driven responses. However, our findings that no interactive effects of H3R and D2R coactivation on several PKA substrates (Fig. S13) were in contrast to the interactive effects on the phosphorylation of Akt and GSK3β (Figs. 5 and S15), suggesting β-arrestin 2 might work as an independent transducer in a Gα-cAMP–PKA-independent manner under these conditions. Previous findings have also indicated that Akt–GSK3β signaling is uncoupled from cAMP–PKA signaling. A cell-permeable cAMP analog (8-Br-cAMP) induces DARPP-32 phosphorylation at Thr^34^ (a readout of PKA signaling) but has no effects on Akt or GSK3β phosphorylation *in vivo* (57). On the other hand, lack of β-arrestin 2 abolishes the effects of D2R activation on Akt but not on DARPP-32 (56). Thus, Akt–GSK3β signaling is likely modulated by cAMP-independent (but β-arrestin 2–dependent) mechanisms. Our findings are in agreement with these studies, with additional selectivity (localizing the interaction to D2-SPNs). Moreover, biased ligands and D2R mutants that signal through G protein–dependent cAMP–PKA or β-arrestin 2–dependent (but G protein–independent) pathways have been identified (83, 97, 98, 99, 100, 101), providing further evidence of β-arrestin 2–dependent G protein–independent pathways. Of note, these conclusions use changes of cAMP–PKA signaling as a readout of G protein function, as is typical in the literature. However, complexes containing GPCR, β-arrestin 1/2, and Gβγ subunits have been described (102, 103, 104). Whether Gβγ subunits play a role in modulating β-arrestin 2–dependent signaling is unknown.

Akt–GSK3β signaling importantly regulates striatal function, especially in D2R-SPNs (56, 82). This pathway has been implicated in preclinical studies of TS and other neuropsychiatric conditions (105, 106), suggesting that physical or functional H3R–D2R interactions could be a locus of intervention in treatment.

Crossantagonism of H3R and D1R at the cellular level is better characterized (21, 40, 41). Here, we used Apo, which activates both D1R and D2R. However, we were unable to replicate the effects of H3R–D1R coactivation described previously (41) on the phosphorylation levels of MSK1 and rpS6 (Figs. S6 and S7). The distinct agonists used (the direct D1R agonist SKF82958 *versus* the D1R–D2R coagonist Apo) and different time points examined (15 min *versus* 30 min after drug administration) may explain this discrepancy.

There are several other limitations in the current study. First, in the Quin experiment, mice were pretreated with reserpine. This strategy is commonly used to isolate postsynaptic D2R effects from those of D2R on DA terminals (21, 107). However, reserpine also depletes other monoamines, including serotonin and norepinephrine, resulting in hypothermia, catalepsy, and hypolocomotion in rodents (108, 109, 110). To control for these effects in behavioral analyses, locomotor baseline was included in the linear mixed effects model as a covariate. We believe that these approaches minimized the impact of confounding drug effects of reserpine. Evidence has shown that different isoforms of D2R are located at different sites, with the short isoform being the predominant DA autoreceptor within the brain, whereas the long isoform is mostly found postsynaptically. These isoforms have different pharmacological properties (19). Isoform-specific ligands might help testing of postsynaptic D2R effects without resorting to the confounding effects of reserpine. However, no such tools have yet been identified.

Second, we used Quin as a D2R agonist to activate D2R signaling. Quin is known to have high affinity for both D2R and D3R. However, it has been shown that D3R has a more restricted distribution in the rodent brain, with little to no expression in the dorsal striatum (111, 112, 113), which is the focus of the current study. So presumably, the drug effects of Quin observed in behavioral and biochemical analyses are largely because of its binding to D2R. Newer drugs like sumanirole shows high selectivity for the D2R subtype over the closely related D3Rs and D4Rs (114, 115), which may be helpful in teasing apart D2R signaling from other subtypes in future experiments. Third, in the current study, drugs were given by systemic administration (Quin and RAMH *via* intraperitoneal injections; reserpine and Apo subcutaneously). H3R is thought to be found only in the central nervous system (14). However, we cannot rule out the possibility of extrastriatal contributors to the observed behavioral and molecular effects. Future work is warranted using local striatal infusion of drugs.

Accumulating evidence has shown that GPCRs form homo-oligomers and hetero-oligomers in the brain (for reviews, see Refs. (28, 116, 117)). These high-order oligomers display distinct functional and biochemical properties from the parental monomers (for reviews, see Refs. (118, 119, 120)). Altered receptor heteromer levels in various brain regions have been shown in animal models of neurological disorders including Alzheimer’s disease (121), Parkinson’s disease (122, 123), Huntington’s disease (71), schizophrenia (53), depression (124, 125, 126, 127), cannabis-induced cognitive impairment (128), and drug abuse (72, 129, 130).

Another limitation of this study is that we did not test directly the requirement of H3R-–D2R heteromers in the behavioral and biochemical outcomes. It remains unknown whether H3R agonism modulates D2R signaling in the heteromers or whether the documented functional interactions arise at some point downstream of the individual receptors. Heteromer-biased ligands will help dissect out distinct signaling transduced by heteromers from that of individual receptors. Such a ligand has been proposed for D1R–D2R heteromers (131), although the specificity of the ligand has been challenged (32). Another approach to investigate specific roles of heteromers is to disrupt the formation of such complexes. Structural studies and computational modeling of GPCRs have identified interfaces in several receptor heteromers, involving transmembrane helices and additional intracellular domains of the protomers (132, 133, 134, 135). Small interfering peptides that target receptor–receptor interactions represent a valuable tool to elucidate role of receptor heteromers in these neurological conditions and may have potential therapeutic benefits in treatment. Indeed, the disruption of receptor heteromers modulates the behavioral phenotypes in some of aforementioned studies (125, 126, 128, 130). In the current work, we demonstrated a functional interaction between H3R and D2R on behavioral outcomes and downstream signaling (mainly Akt–GSK3β in D2-SPNs). Future work will focus on the characterization of interfering peptides that disrupt the H3R–D2R heteromers, and the functional consequences of such manipulations. These efforts may help clarify the role of H3R in modulating the striatum-basal ganglia function, leading to a better understanding of pathophysiology of a range of neuropsychiatric disorders involving the interaction between HA and DA.

## Experimental procedures

### Animals and treatment

All experimental procedures were approved by the Yale University Institutional Animal Care and Use Committee, in accordance with the National Institutes of Health (NIH) Guide for the Care and Use of Laboratory Animals. Male and female C57BL/6J mice were purchased from the Jackson Laboratory (http://jaxmice.jax.org/strain/013636.html). Double-transgenic D1-DARPP-32-FLAG/D2-DARPP-32-Myc were backcrossed to C57BL/6J for at least nine generations and have been described previously; the transgenically expressed FLAG and Myc epitope tags allow dissociable immunostaining of D1R- and D2R-expressing SPN populations (47, 55). All mice were maintained on a 12 h light/dark cycle and used at 3 to 6 months of age.

Quin, RAMH, and reserpine were obtained from Tocris. R-(−)-Apo hydrochloride hemihydrate (Apo) was obtained from Sigma. Quin and RAMH were dissolved in sterile Sal (sodium chloride 0.9%; Hospira) and were injected at 0.5 mg/kg (i.p.) and 45 mg/kg (i.p.), respectively. Reserpine was dissolved in Sal with 0.2% acetic acid and 5.5% glucose (21) and administered at 2 mg/kg (s.c.) 20 h prior to Quin and RAMH treatment. Apo was dissolved in Sal with 0.1% ascorbic acid and administered at 2 mg/kg (s.c.). Dosage of RAMH was chosen as described (41). Dosage of other drugs was determined in pilot work, and corresponding diluents were used as controls.

### Behavioral assessments

Mice were acclimated to the testing room for at least 1 h prior to experimentation. Mice were placed in activity chambers (OmniTech Electronics) for 30 min for habituation, followed by drug treatment. Activity (beam break) was monitored for 45 min after drug administration using the Fusion software (OmniTech Electronics) (24, 41). Total distance traveled, ambulatory activity (beam breaks because of ambulation), and stereotypic activity (repeated breaks of the same set of beams) were automatically scored.

### Immunohistochemistry

Thirty minutes after drug administration, mice were anesthetized by intraperitoneal injection of ketamine (100 mg/kg) with xylazine (10 mg/kg) and transcardially perfused with cold 4% paraformaldehyde in 1× PBS (pH 7.4) with 1 mM NaF. Brains were fixed overnight in 4% paraformaldehyde at 4 °C, followed by equilibrating in 30% sucrose for 48 h at 4 °C. Striatal slices were cut at 20 μm using a Leica CM3050S cryostat (Leica). Slices were stored in a cryoprotectant solution (30% glycerin, 30% ethylene glycol in 1× PBS [pH 7.4] with 1 mM NaF) at −20 °C until use. Brain sections from D1-FLAG/D2-Myc double transgenic mice were used to label D1R- and D2R-expressing SPNs (47, 55). Slices were washed 3 × 10 min in 1× PBS (pH 7.4) to remove cryoprotectant, followed by incubation in freshly prepared 0.1% Sudan Black (in 70% ethanol) for 10 min at room temperature (RT) to quench autofluorescence. Slices were washed 3 × 10 min in 70% ethanol and 3 × 10 min in 1× PBS. Slices were blocked in 1× PBS + 0.3% Triton X-100 supplemented with 5% donkey serum (Jackson Immunoresearch) for 1 h at RT, and in addition blocked in Mouse-on-Mouse reagent (Vector Laboratories), following the manufacturer’s instructions. Slices were then incubated with primary antibodies (Table S1) in blocking buffer overnight at 4 °C. The next day, slices were washed 3 × 10 min in blocking buffer and then incubated with fluorophore-conjugated secondary antibodies (Table S1) for 1 h at RT. After 3 × 10 min washes in 1× PBS, slices were mounted in Vectashield HardSet Mounting Medium (Vector Laboratories), coverslipped, sealed with nail polish, and stored at 4 °C.

Confocal imaging was performed by sequential scanning at 40× using an Olympus Fluoview FV-1000 confocal microscope equipped with 473, 559, and 635 nm lasers. Images were acquired with a Kalman filter at a scan rate of 4 μs/pixel. Six Z-stacks were collected with a step size of 1 μm for each field of view.

### *In situ* PLA

Free-floating brain sections from C57BL/6J mice were used. Sections were incubated with pairs of primary antibodies (Table S1): H3R–D1R, H3R–D2R, D1R–σ1R, D1R–σ2R, D2R–σ1R, and D2R–σ2R, following the standard immunohistochemistry procedures described previously. Negative controls with only one of the antibodies in the pairs were also employed. Receptor heteromers were detected using Duolink *In Situ* Red Starter Kit with Goat/Rabbit probes (Sigma–Aldrich), following the manufacturer’s protocol. Anti–guinea pig probe was not commercially available, donkey anti–guinea pig IgG (Jackson ImmunoResearch Laboratories) was conjugated to the PLUS oligo using Duolink *In Situ* Probemaker PLUS kit (Sigma–Aldrich), following the manufacturer’s protocol. Briefly, after incubation with primary antibodies, slices were washed with buffer A in the kit, and incubated with probes (oligonucleotide-conjugated second antibodies: antigoat PLUS with anti-rabbit MINUS or anti–guinea pig PLUS with anti-rabbit MINUS) for 1 h at 37 °C in a humidity chamber. After two washes with buffer A, sections were incubated with the ligation solution for 30 min at 37 °C. The sections were washed with buffer A twice and incubated with the amplification solution for 100 min at 37 °C. After amplification, sections were washed with buffer B twice and a final wash with 0.01× buffer B, and then mounted onto microscope slides with mounting medium containing 4′,6-diamidino-2-phenylindole (Sigma). The slides were coverslipped and sealed with nail polish and temporarily stored at 4 °C before confocal microscope analysis.

Confocal images were acquired in the dorsal striatum by sequential scanning at 60× using an Olympus Fluoview FV-1000 confocal microscope equipped with 405 and 559 nm lasers. Eleven Z-stacks with a step size of 0.5 μm were collected for each field of view.

### Image processing and quantification

Automated quantitation of confocal images was achieved using Fiji ImageJ from the NIH (https://imagej.net/Fiji/Downloads) with batch processing. An object-based approach was used to identify objects (cells) in each individual channel, for neuronal marker immunostaining (FLAG epitope tag for D1R-SPNs; Myc epitope tag for D2R-SPNs) and immunostaining of signaling molecules (Akt, GSK3β, MSK1, and rpS6). Objects were identified using the 3D-Object counter plug-in using fixed thresholds for each channel, determined using autothreshold, averaged across images, and then kept constant for the same target. Expression of signaling molecules in D1R- and D2R-SPNs was calculated by the overlapping of objects (cells) identified in both channels; double-positive cell numbers were normalized to total D1R- or D2R-SPNs to get the proportion of D1R- and D2R-SPNs that also expressed one of the signaling molecules. The number of total cells positive for each signaling molecule was also counted. In addition to cell numbers, staining immunofluorescence intensity (density in 3D cells) was also scored for all three populations (double positive for signaling molecule and D1R; double positive for signaling molecule and D2R; and total positive for signaling molecule). Confocal images were acquired in three batches. All the values (proportion of cell number and staining density) were normalized to the mean values of the control groups in each batch to reduce batch effects.

For the PLA experiments, total number of PLA signals (*red puncta*) and cells (*blue nuclei*) were quantified on the maximum projections of each image stack using the Andy’s Algorithm (136). Briefly, channels were split and processed separately. Images of nuclei (*blue*) went through background subtraction, segmentation, and the watershed algorithms to obtain the binary images containing individual nuclei. Size exclusion was applied to remove smaller nuclei from glia cells. PLA signal images were thresholded to obtain individual puncta. The number of *red puncta* per nucleus was used as an index of the density of receptor heteromers.

### Coimmunoprecipitation of receptor heteromers and Akt-containing complex

After treatment, mice were sacrificed by cervical dislocation, and striata were rapidly dissected out and snap-frozen on dry ice. Tissues were then homogenized in lysis buffer: 20 mM Tris–HCl, pH 7.4, 0.5% NP-40, supplemented with complete phosphatase and protease inhibitors (Roche), and spun at 1000*g* for 10 min to obtain the soluble fraction. Protein concentrations were measured using a bicinchoninic acid protein assay kit (Pierce). For coimmunoprecipitation of H3R-containing receptor heteromers, lysates (500 μg) were first precleared with protein A/G-agarose beads (Santa Cruz Biotechnology) for 2 h at 4 °C, and mixed with anti-D1R, anti-D2R, anti-H3R antibodies (Table S1) or isotype IgG controls overnight at 4 °C as described (137). On the second day, protein A/G-agarose was added to the antibody-bound complex for 4 h at 4 °C, and precipitates were washed three times with lysis buffer and eluted in heated Laemmli sample buffer (Bio-Rad).

For coimmunoprecipitation of Akt-containing signaling complex, lysates were precleared with protein A/G-agarose beads and mixed with Sepharose bead conjugated-Akt antibody (Table S1) overnight at 4 °C as described (56). After washes, immune complex was eluted in heated Laemmli sample buffer.

### Immunoblotting

Eluted immune complex from coimmunoprecipitation experiments or total protein lysates (in Laemmli sample buffer) were heated for 5 min at 100 °C on a heating block. Then samples were resolved on 8% SDS-PAGE. Proteins were transferred onto nitrocellulose membranes (Bio-Rad), blocked in blocking buffer (Tris-buffered Sal + 0.1% Tween-20 + 5% bovine serum albumin) for 1 h at RT. Membranes were then incubated with specific primary antibodies (as listed in Table S1) in blocking buffer overnight at 4 °C. On the second day, membranes were incubated with peroxidase-conjugated secondary antibodies (Table S1) for 2 h at RT. Immunoreactivity was developed using a Chemiluminescent Substrate kit (Thermo Fisher) and visualized using ChemiDoc XRS+ system (Bio-Rad). All densitometric bands were quantified using ImageJ (NIH).

### Statistical analysis

All data are expressed as means ± SD. Statistical analyses were performed using SPSS Statistics 28 (IBM) or GraphPad Prism 9.3 (GraphPad Software, Inc). Significance (*p* < 0.05) was determined by two-tailed unpaired *t* test, two-way ANOVA, two-way analysis of covariance, or linear mixed effects model with baseline activity as a covariate, as indicated for each experiment. Where statistical effects were significant, post hoc tests were carried out using Tukey or Bonferroni tests for multiple group comparisons. See Table S2 for descriptive statistics and detailed statistical analyses.

## Data availability

All data described in this work are contained in this article and in the accompanying supporting information.

## Supporting information

This article contains supporting information (49, 50, 51, 52).

## Conflict of interest

Dr Pittenger has served as a consultant and received research funding in the past year for Biohaven Pharmaceuticals, Transcend Therapeutics, Ceruvia Lifesciences, Freedom Biosciences, and Nobilis Therapeutics, and royalties from Oxford University Press, all for work unrelated to the current results. He is an inventor on a patent application related to the use of neurofeedback and of psychedelic drugs for the treatment of obsessive compulsive disorder, also unrelated to this work, Dr Xu reports no competing interests. The authors declare that they have no conflicts of interest with the contents of this article.
