# Supplementary material for: The histamine H3 receptor modulates dopamine D2 receptor–dependent signaling pathways and mouse behaviors
Source: J Biol Chem. 2023 Mar 4;299(4):104583. doi: 10.1016/j.jbc.2023.104583 (PMC10139999; doi:10.1016/j.jbc.2023.104583)
Supplement: Supporting Information [file mmc1.docx]

**Supporting Information**

**The histamine H3 receptor modulates dopamine D2 receptor-dependent signaling pathways and mouse behaviors**

Jian Xu, Ph.D.^1,*^, Christopher Pittenger, M.D., Ph.D.^1-6*^

Departments of ^1^Psychiatry and ^2^Psychology, ^3^Child Study Center, ^4^Interdepartmental Neuroscience Program, ^5^Wu-Tsai Institute, and ^6^Center for Brain and Mind Health, Yale University.

^*^Address correspondence to:

- Jian Xu, Yale University School of Medicine, 34 Park Street rm W306, New Haven, CT 06519. Email: [jian.xu@yale.edu](mailto:jian.xu@yale.edu)
- Christopher Pittenger, Yale University School of Medicine, 34 Park Street rm 335, New Haven, CT 06519. Phone: 203-974-7675. Email: [christopher.pittenger@yale.edu](mailto:christopher.pittenger@yale.edu).

**Material included:** Supplementary materials and methods, Figure S1-S18, Table S1-S3.

**Supplementary Materials and methods**

*Validation of phospho-specific antibodies for immunohistochemistry*

To verify that the phospho-specific antibodies (**Table S1**) do not stain non-phospho isoforms of corrresponding targets, brain sections were pretreated with calf intestinal alkaline phosphatase (Quick CIAP, New England BioLabs) following manufacturer’s protocol. Briefly, brain sections were washed 3× 10 min in 1× TBS and then blocked and permeabilized in blocking buffer: 1× TBS + 0.3% Triton X-100 + 5% normal donkey serum for 1 h at RT. After blocking, sections were washed 3× 10 min in 1× TBS and incubated in 1× CutSmart buffer + 100 U/mL CIAP for 4 h at 37°C in a humidity chamber with gentle shaking. Several sections were incubated with 1× CutSmart buffer alone without CIAP as controls. After dephosphorylation, sections were washed 3× 10 min in 1× TBS, followed by standard immunohistochemistry procedures.


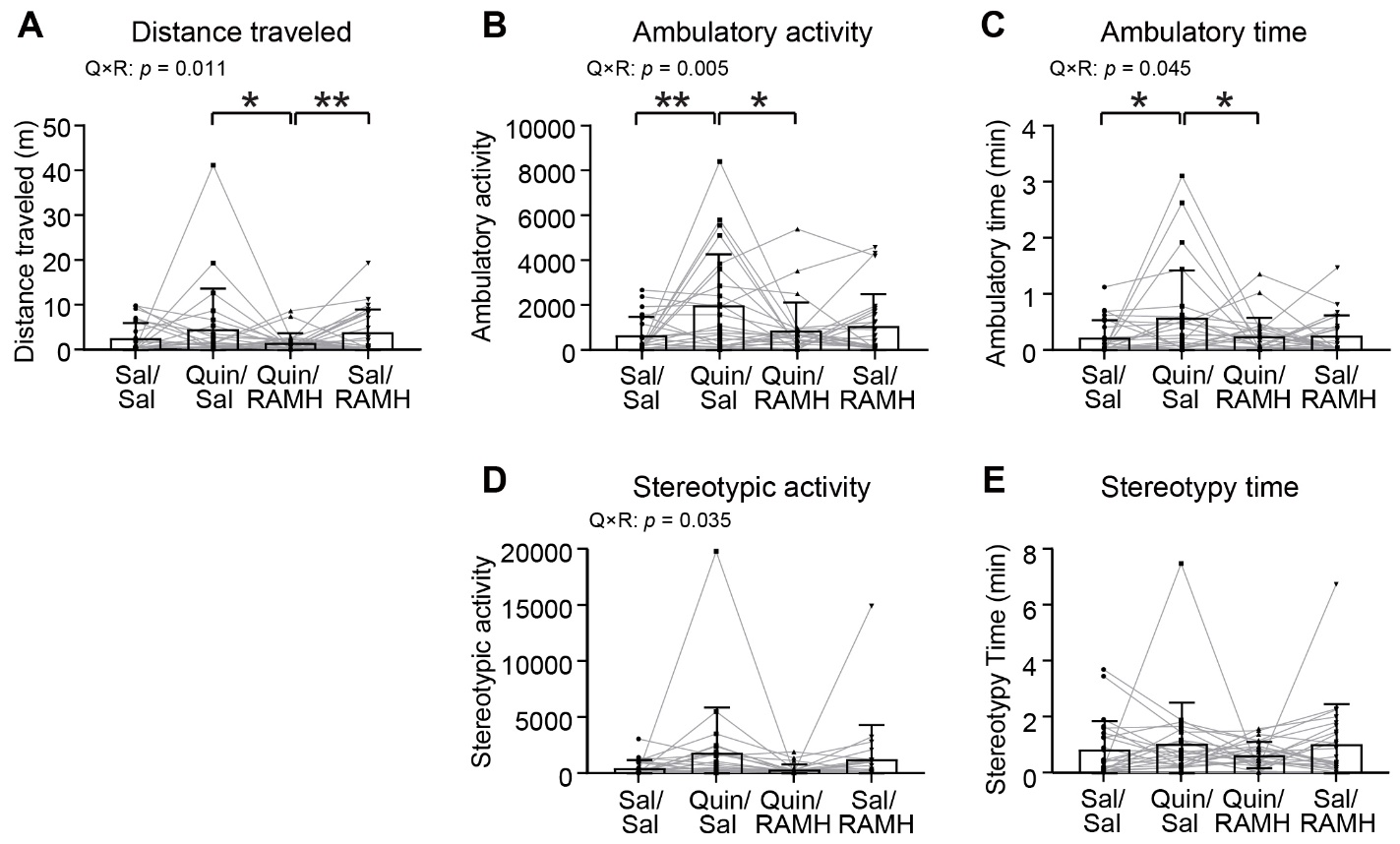


**Figure S1. H3R agonist co-administration attenuates D2R agonist-induced locomotor and stereotypic behavior.** Male and female D1-FLAG/D2-Myc mice received reserpine (2 mg/kg, s.c.) 20 h prior to drug administration. Mice were placed in an activity chamber for 30 min, received injections of saline (Sal) or RAMH (45 mg/kg, i.p.), followed by Sal or quinpirole (Quin, 0.5 mg/kg, i.p.), and then monitored for 45 min. General locomotor activity including distance traveled (*A*), ambulatory activity counts (*B*) and ambulatory activity time (*C*) as well as stereotypic activity counts (*D*) and stereotypy time (*E*) were counted. Raw values for those in Figure 1 were plotted. ***A***, distance traveled. Quin × RAMH interaction, F(1, 31.36) = 7.351, *p* = 0.011; main effect of Quin, F(1, 32.72) = 0.003, *p* = 0.955; main effect of RAMH, F(1, 34.40) = 1.921, p = 0.175. ***B***, ambulatory activity counts. Quin × RAMH interaction: F(1, 33.00) = 9.071, *p* = 0.005; main effect of Quin: F(1, 35.81) = 5.092, p = 0.030; main effect of RAMH: F(1, 50.83) = 2.745, *p* = 0.104. ***C***, ambulatory activity time. Quin × RAMH interaction: F(1, 25.70) = 4.424, *p* = 0.045; main effect of Quin: F(1, 32.39) = 3.579, p = 0.067; main effect of RAMH: F(1, 33.64) = 3.474, p = 0.071. ***D***, stereotypic activity counts. Quin × RAMH interaction: F(1, 40.37) = 4.750, *p* = 0.035; main effect of Quin: F(1, 44.36) = 0.230, *p* = 0.634; main effect of RAMH: F(1, 43.27) = 0.572, *p* = 0.454. ***E***, stereotypy time. Quin × RAMH interaction: F(1, 41.39) = 1.046, *p* = 0.312; main effect of Quin: F(1, 57.29) = 0.152, *p* = 0.698; main effect of RAMH: F(1, 55.85) = 0.137, *p* = 0.713. All values are expressed as mean ± SEM. Statistical analysis was performed using a linear mixed effects model with baseline activity as a covariate in SPSS 28. Where significant drug interactions or main effects were detected, multiple comparisons were conducted using post hoc Bonferroni test. **p* < 0.05, ***p* < 0.01, n = 24 each group (9 male and 15 female mice).


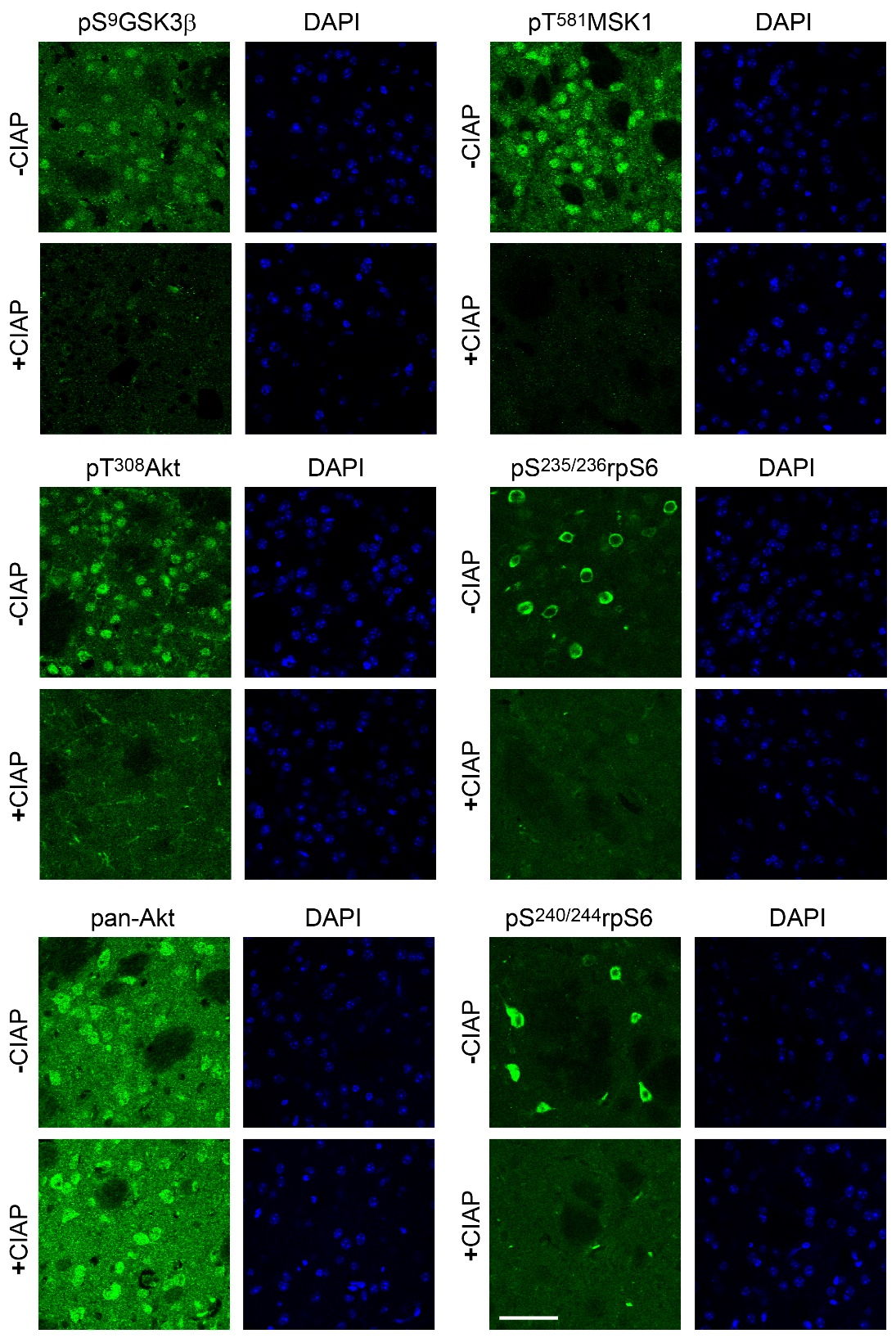


**Figure S2. Validation of phospho-specific antibodies used in immunohistochemcial staining.** Mouse striatal sections were pretreated with calf intestinal alkaline phosphatase (+CIAP) or buffer alone (-CIAP), followed by standard IHC protocol. Phospho-antibodies (**Table S1**) labeled corresponding targets in buffer-treated sections but not CIAP-treated brain sections, indicating these antibodies were specific towards phospho-isoforms of each target as predicted. Cell nuclei were counterstained with DAPI (blue). Scale bar: 50 μm.


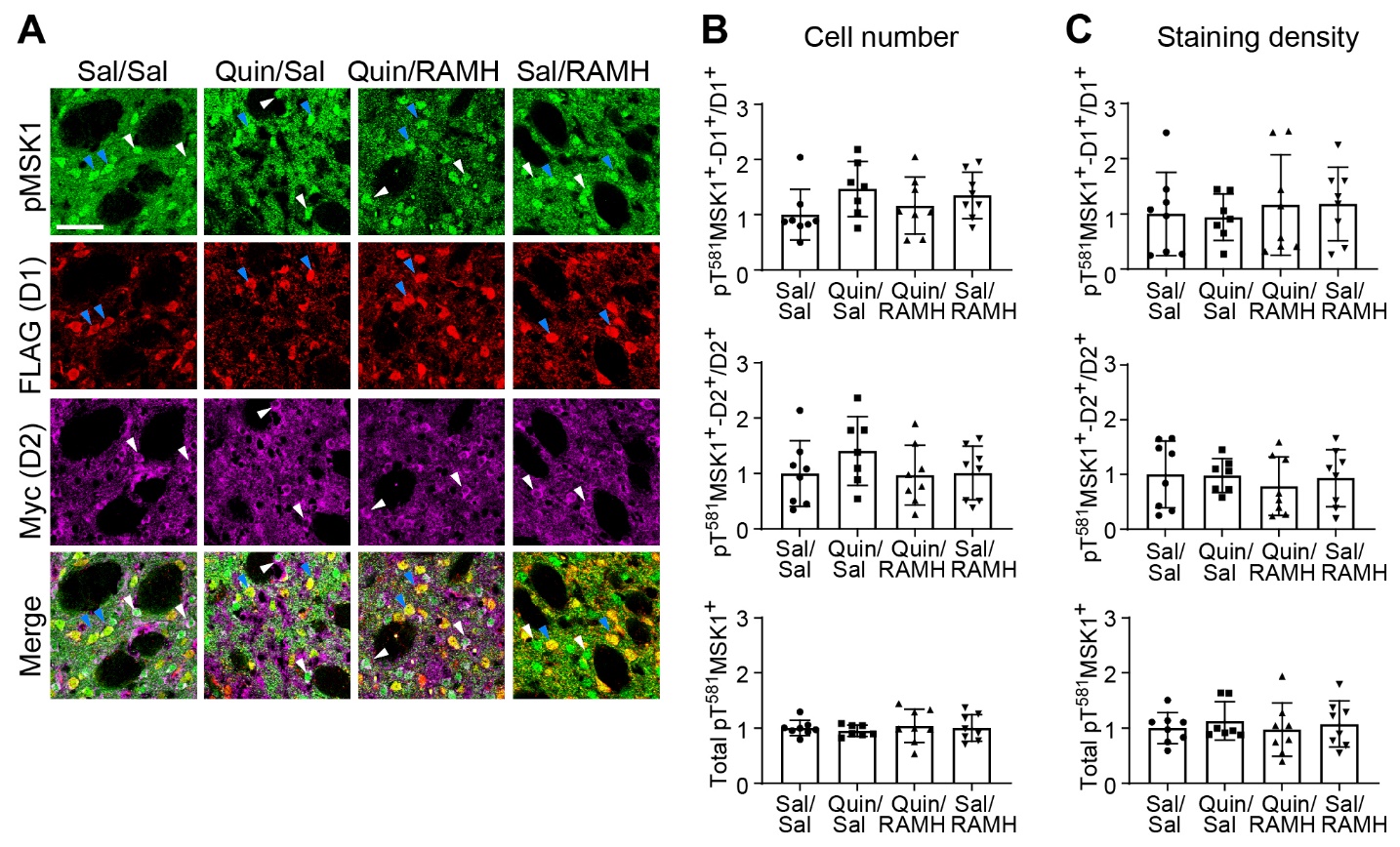


**Figure S3. H3R and D2R co-activation does not alter phosphorylation of MSK1 in D1R- or D2R-SPNs.** Male and female D1-FLAG/D2-Myc mice received reserpine (2 mg/kg, s.c.) 20 h prior to drug administration. Mice received injections of saline (Sal) or RAMH (45 mg/kg, i.p.), followed by Sal or quinpirole (Quin, 0.5 mg/kg, i.p.), and anesthetized 30 min after treatment. ***A***, Representative images of pT^581^ MSK1 immunostaining in D1R- and D2R-SPNs. Blue and white arrow heads indicate D1R-SPNs (labeled by the FLAG epitope) and D2R-SPNs (labeled by the Myc epitope), respectively. Merged images were obtained by overlaying 3 channels from the same field of view. Scale bar: 50 μm. ***B***, The proportion of pT^581^ MSK1-positive cells in D1R- and D2R-SPNs and the total number of pT^581^ MSK1-positive cells. ***C***, Staining density of pT^581^ MSK1-positive cells in D1R- and D2R-SPNs and the total fluorescence density of pT^581^ MSK1-positive cells. All values are expressed as mean ± SEM. Statistical analysis was performed using two-way ANOVAs in GraphPad Prism 9. No drug interactions or main effects of drugs were found. See **Table S2** for additional statistical analyses. n = 8 each group.


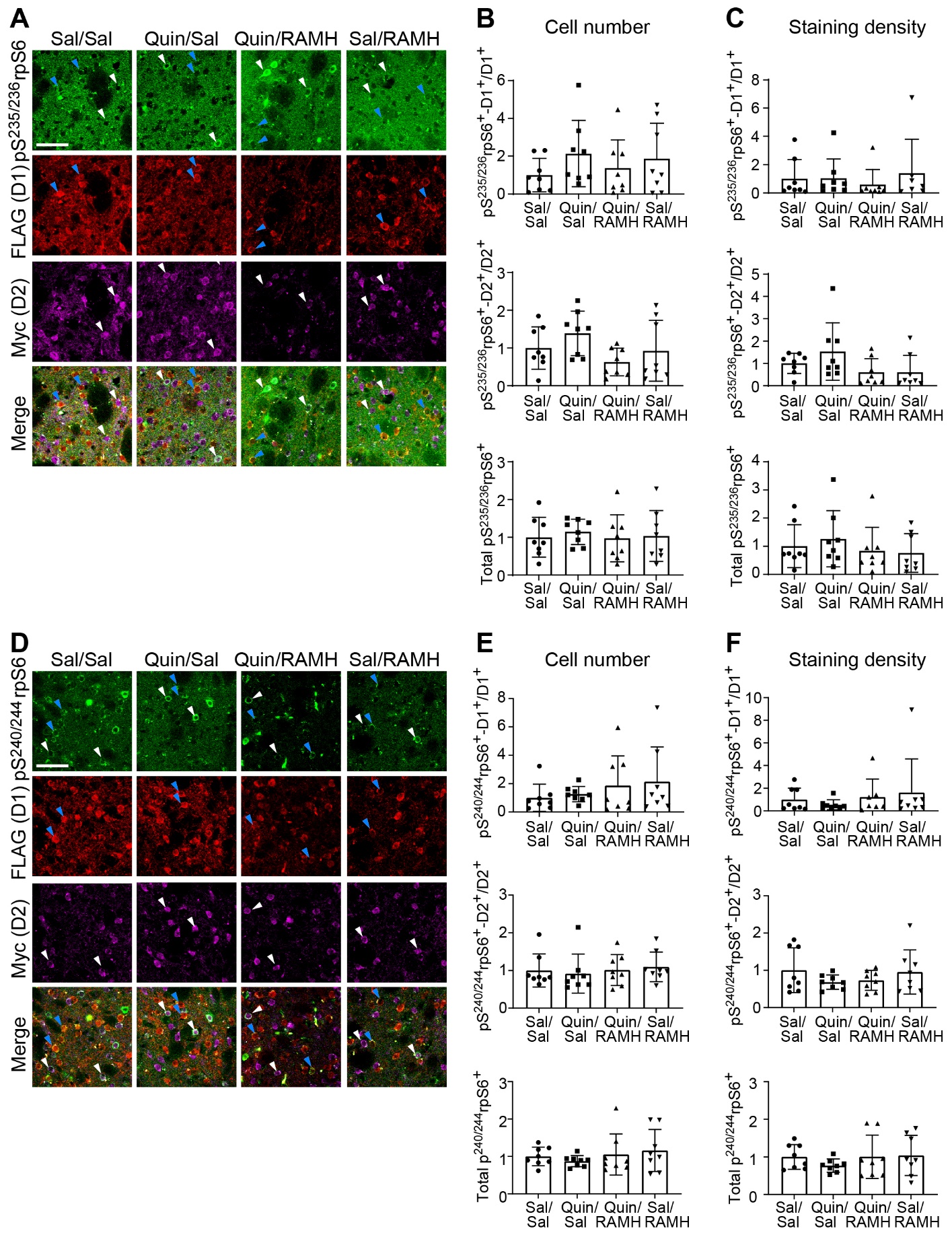


**Figure S4. H3R and D2R co-activation does not alter phosphorylation of rpS6 in D1R- or D2R-SPNs.** Male and female D1-FLAG/D2-Myc mice received reserpine (2 mg/kg, s.c.) 20 h prior to drug administration. Mice received injections of saline (Sal) or RAMH (45 mg/kg, i.p.), followed by Sal or quinpirole (Quin, 0.5 mg/kg, i.p.), and anesthetized 30 min after treatment. ***A***, Representative images of pS^235/236^ rpS6 immunostaining in D1R- and D2R-SPNs. Blue and white arrow heads indicate D1R-SPNs (labeled by the FLAG epitope) and D2R-SPNs (labeled by the Myc epitope), respectively. Merged images were obtained by overlaying 3 channels from the same field of view. Scale bar: 50 μm. ***B***, The proportion of pS^235/236^ rpS6-positive cells in D1R- and D2R-SPNs and the total number of pS^235/236^ rpS6-positive cells. ***C***, Staining density of pS^235/236^ rpS6-positive cells in D1R- and D2R-SPNs and the total fluorescence density of pS^235/236^ rpS6-positive cells. ***D***, Representative images of pS^240/244^ rpS6 immunostaining in D1R- and D2R-SPNs. Blue and white arrow heads indicate D1R-SPNs (labeled by the FLAG epitope) and D2R-SPNs (labeled by the Myc epitope), respectively. Merged images were obtained by overlaying 3 channels from the same field of view. Scale bar: 50 μm. ***E***, The proportion of pS^240/244^ rpS6-positive cells in D1R- and D2R-SPNs and the total number of pS^240/244^ rpS6-positive cells. ***F***, Staining density of pS^240/244^ rpS6-positive cells in D1R- and D2R-SPNs and the total fluorescence density of pS^240/244^ rpS6-positive cells. All values are expressed as mean ± SEM. Statistical analysis was performed using two-way ANOVAs in GraphPad Prism 9. No drug interactions or main effects of drugs were found. See **Table S2** for additional statistical analyses. n = 8 each group.


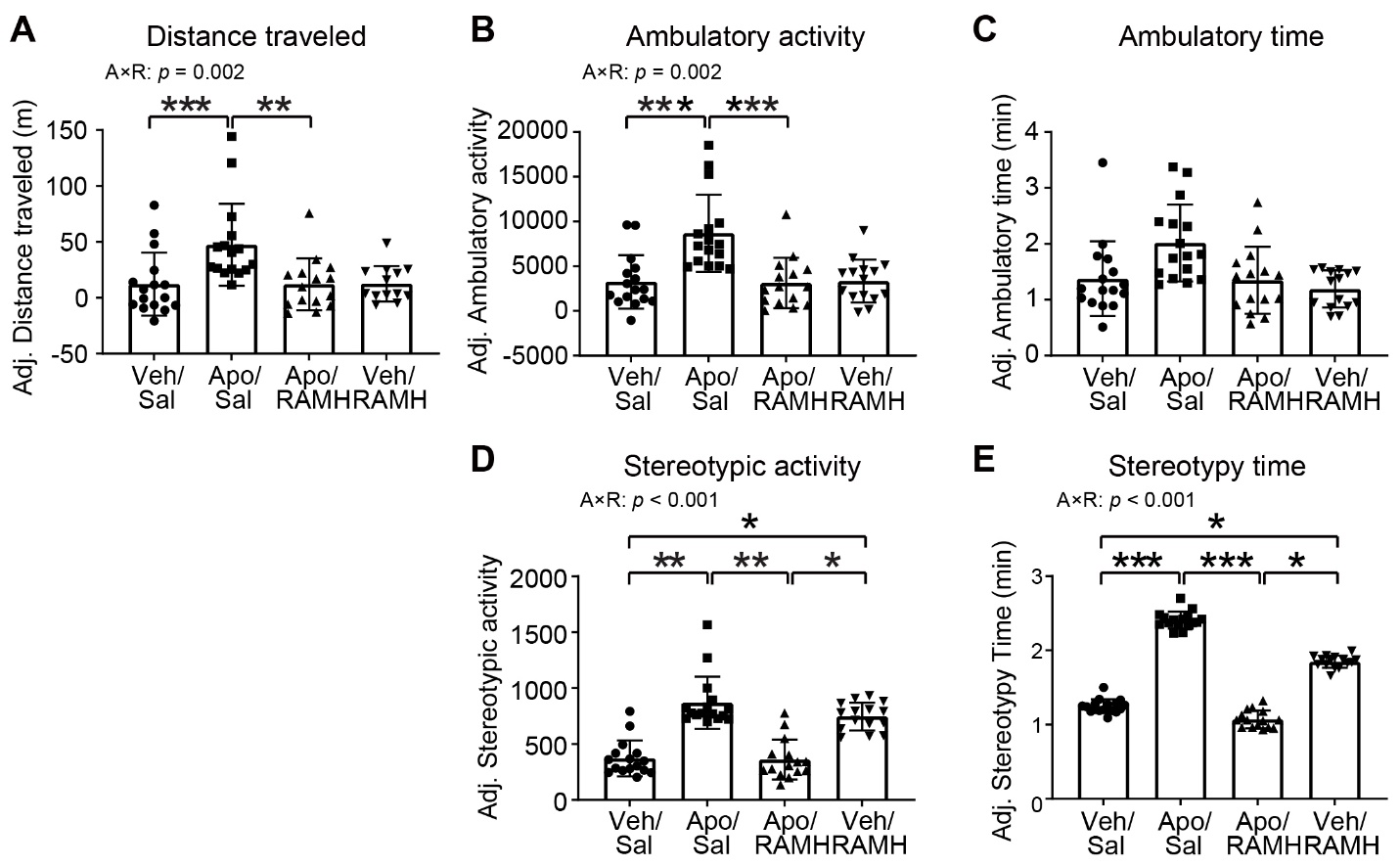


**Figure S5. H3R agonist co-administration attenuates apomorphine-induced locomotor and stereotypic behavior.** Male and female D1-FLAG/D2-Myc mice were placed in activity chambers for 30 min, received injections of saline (Sal) or RAMH (45 mg/kg, i.p.), followed by Vehicle (Veh) or apomorphine (Apo, 2 mg/kg, s.c.), and then monitored for 45 min. General locomotor activity including distance traveled (*A*), ambulatory activity counts (*B*) and ambulatory activity time (*C*) as well as stereotypic activity counts (*D*) and stereotypy time (*E*) were counted. Covariate-adjusted values were plotted for clearer drug effects, while statistical analysis was performed using raw values. ***A***, distance traveled. Apo × RAMH interaction, F(1, 55) = 10.175, *p* = 0.002, *η^2^_p_* = 0.156. ***B***, ambulatory activity counts. Apo × RAMH interaction: F(1, 57) = 8.147, *p* = 0.006, *η^2^_p_* = 0.125; main effect of Apo: F(1, 57) = 3.996, *p* = 0.050, *η^2^_p_* = 0.066. ***C***, ambulatory activity time. Apo × RAMH interaction: F(1, 57) = 2.181, *p* = 0.145. ***D***, stereotypic activity counts. Apo × RAMH interaction: F(1, 57) = 14.953, *p* < 0.001, *η^2^_p_* = 0.208. ***E***, stereotypy time. Apo × RAMH interaction: F(1, 57) = 22.378, *p* < 0.001, *η^2^_p_* = 0.282. All values are expressed as mean ± SEM. Statistical analysis was performed using two-way ANCOVAs with baseline activity as a covariate in GraphPad Prism 9. See **Table S2** for additional statistical analyses. Where significant drug interactions or main effects were detected, multiple comparisons were conducted using post hoc Bonferroni test. **p* < 0.05, ***p* < 0.01, ****p* < 0.01, n = 16 for Veh/Sal and Apo/Sal groups; n = 15 for Apo/RAMH and Veh/RAMH groups.


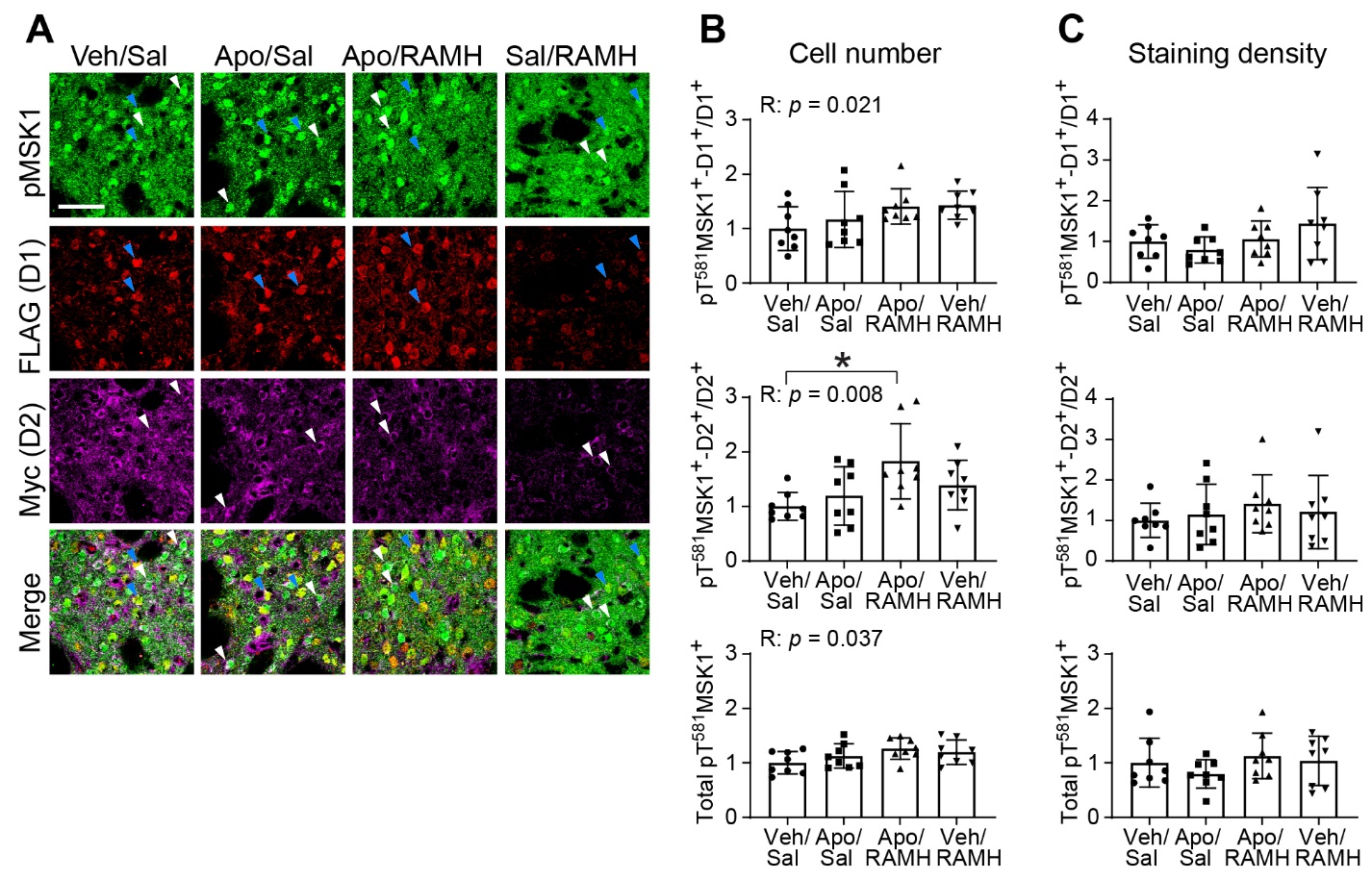


**Figure S6. H3R and D1R/D2R co-activation show subtle effects on phosphorylation of MSK1 in D1R- and D2R-SPNs.** Male and female D1-FLAG/D2-Myc mice received injections of saline (Sal) or RAMH (45 mg/kg, i.p.), followed by vehicle (Veh) or apomorphine (Apo, 2 mg/kg, s.c.), and anesthetized 30 min after treatment. ***A***, Representative images of pT^581^ MSK1 immunostaining in D1R- and D2R-SPNs. Blue and white arrow heads indicate D1R-SPNs (labeled by the FLAG epitope) and D2R-SPNs (labeled by the Myc epitope), respectively. Merged images were obtained by overlaying 3 channels from the same field of view. Scale bar: 50 μm. ***B***, The proportion of pT^581^ MSK1-positive cells in D1R- and D2R-SPNs and the total number of pT^581^ MSK1-positive cells. Upper panel, main effect of RAMH: F (1, 28) = 5.977, *p* = 0.021, *η^2^_p_* = 0.176. Middle panel, main effect of RAMH: F (1, 28) = 8.144, *p* = 0.008, *η^2^_p_* = 0.225. Lower panel, F (1, 28) = 4.786, *p* = 0.037, *η^2^_p_* = 0.146. ***C***, Staining density of pT^581^ MSK1-positive cells in D1R- and D2R-SPNs and the total fluorescence density of pT^581^ MSK1-positive cells. All values are expressed as mean ± SEM. No drug interactions or main effects of drugs were found. Statistical analysis was performed using two-way ANOVAs in GraphPad Prism 9. See **Table S2** for additional statistical analyses. Where significant main effects were detected, multiple comparisons were conducted using post hoc Bonferroni test. **p* < 0.05, n = 8 each group.


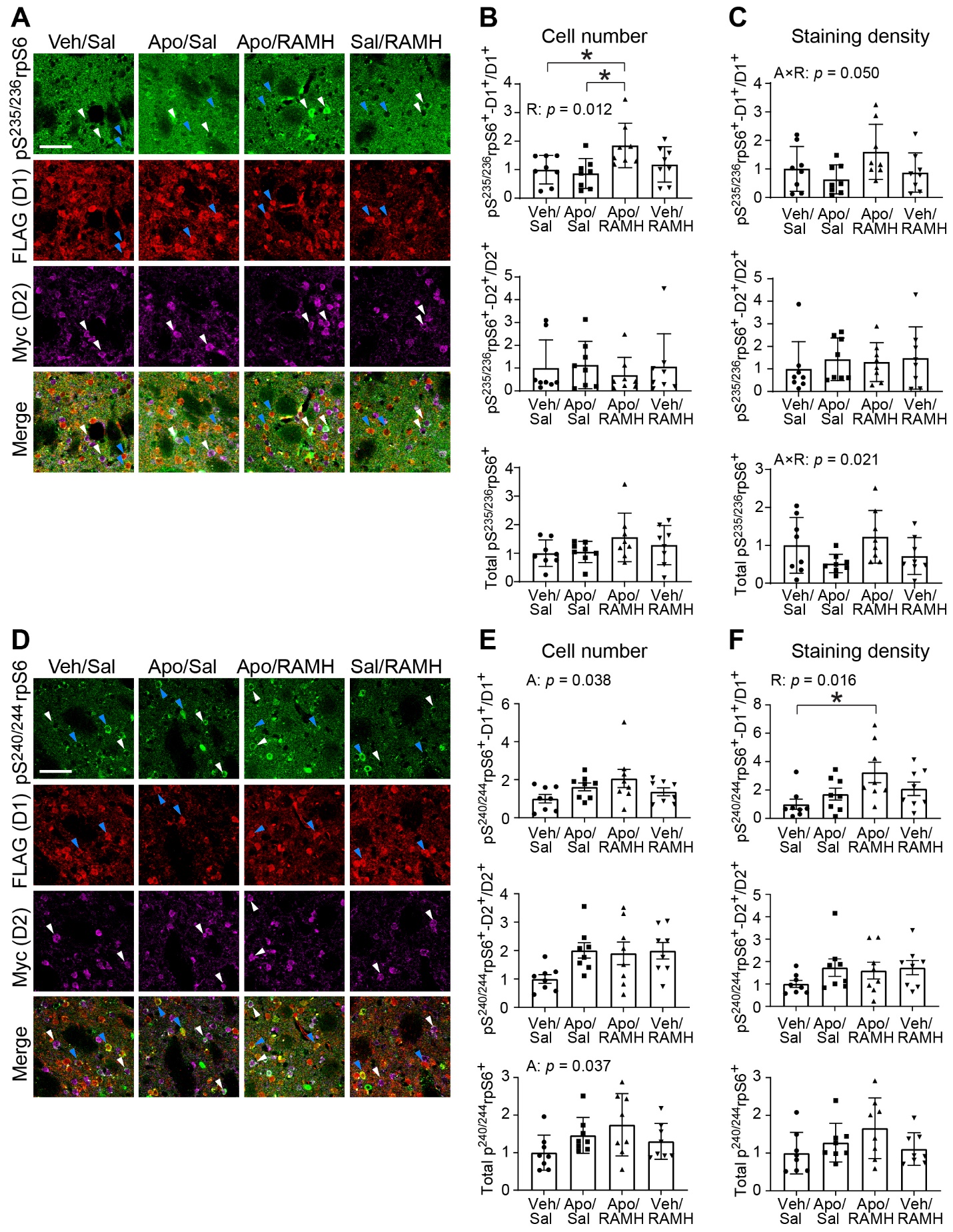


**Figure S7. H3R and D1R/D2R co-activation show subtle effects on phosphorylation of rpS6 in D1R-SPNs.** Male and female D1-FLAG/D2-Myc mice received injections of saline (Sal) or RAMH (45 mg/kg, i.p.), followed by vehicle (Veh) or apomorphine (Apo, 2 mg/kg, s.c.), and anesthetized 30 min after treatment. ***A***, Representative images of pS^235/236^ rpS6 immunostaining in D1R- and D2R-SPNs. Blue and white arrow heads indicate D1R-SPNs (labeled by the FLAG epitope) and D2R-SPNs (labeled by the Myc epitope), respectively. Merged images were obtained by overlaying 3 channels from the same field of view. Scale bar: 50 μm. ***B***, The proportion of pS^235/236^ rpS6-positive cells in D1R- and D2R-SPNs and the total number of pS^235/236^ rpS6-positive cells. Upper panel, main effect of RAMH: F (1, 28) = 7.213, *p* = 0.012, *η^2^_p_* = 0.205. ***C***, Staining density of pS^235/236^ rpS6-positive cells in D1R- and D2R-SPNs and the total fluorescence density of pS^235/236^ rpS6-positive cells. Upper panel, Quin × RAMH interaction: F (1, 28) = 4.208, p = 0.050, *η^2^_p_* = 0.131. Lower panel, Quin × RAMH interaction: F (1, 28) = 5.965, *p* = 0.021, *η^2^_p_* = 0.176. ***D***, Representative images of pS^240/244^ rpS6 immunostaining in D1R- and D2R-SPNs. Blue and white arrow heads indicate D1R-SPNs (labeled by the FLAG epitope) and D2R-SPNs (labeled by the Myc epitope), respectively. Merged images were obtained by overlaying 3 channels from the same field of view. Scale bar: 50 μm. ***E***, The proportion of pS^240/244^ rpS6-positive cells in D1R- and D2R-SPNs and the total number of pS^240/244^ rpS6-positive cells. Upper panel, main effect of Apo: F (1, 28) = 4.727, *p* = 0.038, *η^2^_p_* = 0.144. Lower panel, main effect of Apo: F (1, 28) = 4.790, *p* = 0.037, *η^2^_p_* = 0.146. ***F***, Staining density of pS^240/244^ rpS6-positive cells in D1R- and D2R-SPNs and the total fluorescence density of pS^240/244^ rpS6-positive cells. Upper panel, main effect of RAMH: F (1, 28) = 6.630, *p* = 0.016, *η^2^_p_* = 0.191. All values are expressed as mean ± SEM. Statistical analysis was performed using two-way ANOVAs in GraphPad Prism 9. See **Table S2** for additional statistical analyses. Where significant main effects were detected, multiple comparisons were conducted using post hoc Bonferroni test. **p* < 0.05, n = 8 each group.


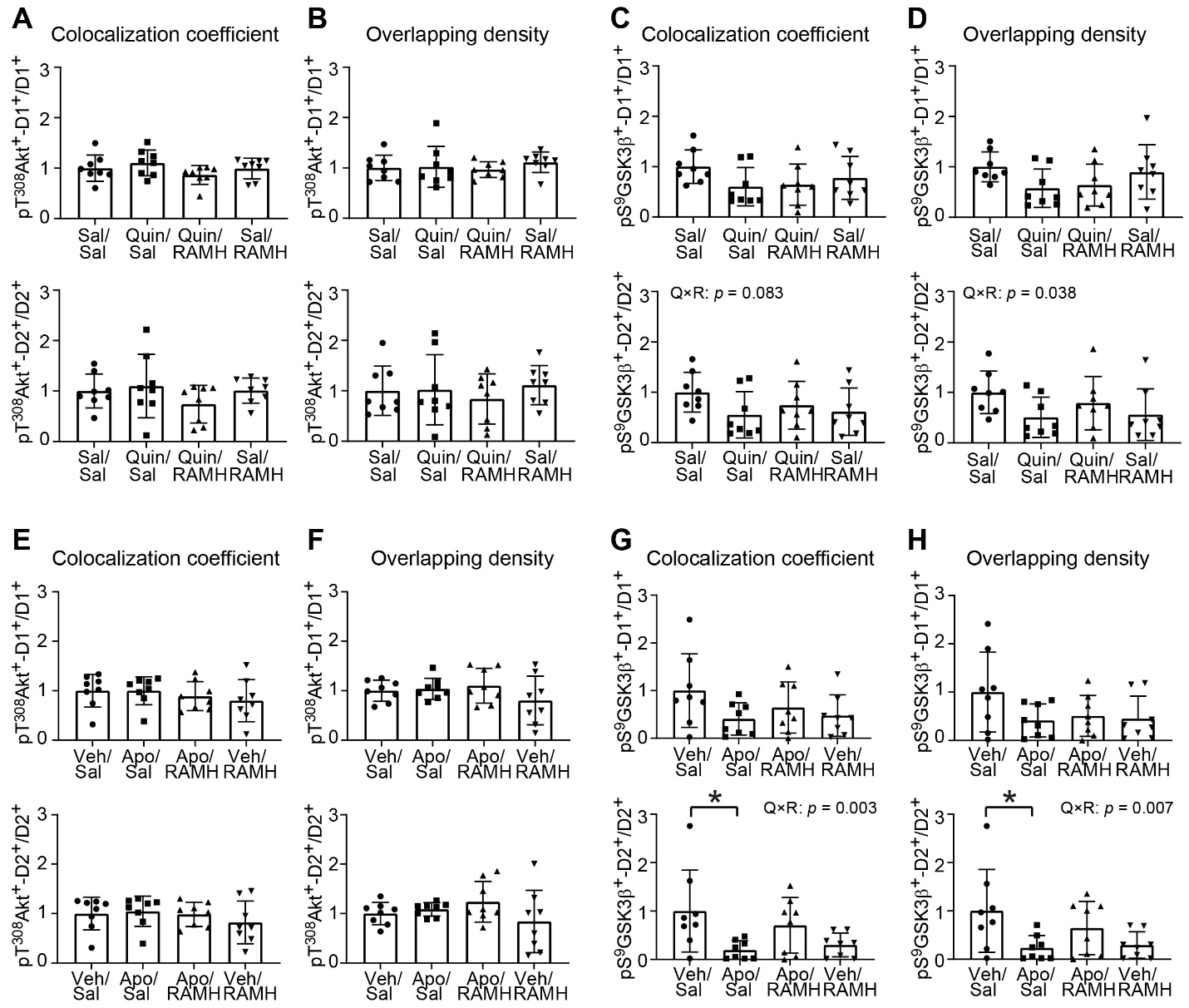


**Figure S8. Modulation of GSK3β by H3R-D2R interaction in D2R-SPNs.** The same set of confocal images from Quin-RAMH (**Fig. 2**) and Apo-RAMH (**Fig. 3**) treatments were reanalyzed using a intensity-based approach. Expression of siganling molecules in D1R- or D2R-SPNs was indicated using Manders’ colocalization coefficient (MCC) as a measure of the degree of pixel overlapping between image channels. The density of overlapping pixels was also quantified. Values were normalized to the control groups in each graph. A-D: Quin-RAMH treatment; E-H: Apo-RAMH treatment. ***A*** and ***E***, Colocalization coefficient of pT^308^ Akt with D1 (FLAG epitope) or D2 (Myc epitope). ***B*** and ***F***, Density of pT^308^ Akt staining within D1R- or D2R-SPNs. ***C*** and ***G***, Colocalization coefficient of pS^9^ GSK3β with D1 (FLAG epitope) or D2 (Myc epitope). Lower panels, Quin × RAMH interaction in D2R-SPNs: F (1, 28) = 3.240, *p* = 0.083; Apo × RAMH interaction in D2R-SPNs: F (1, 28) = 10.250, *p* = 0.003, *η^2^_p_* = 0.268. ***D*** and ***H***, Density of pS^9^ GSK3β staining within D1R- or D2R-SPNs. Lower panels, Quin × RAMH interaction in D2R-SPNs: F (1, 28) = 4.720, *p* = 0.038, *η^2^_p_* = 0.144; Apo × RAMH interaction in D2R-SPNs: F (1, 28) = 8.408, *p* = 0.007, *η^2^_p_* = 0.231. All values are expressed as mean ± SEM. Statistical analysis was performed using two-way ANOVAs in GraphPad Prism 9. See **Table S2** for additional statistical analyses. Where significant drug interactions or main effects were found, multiple comparisons were conducted using post hoc Tukey test. **p* < 0.05, n = 8 each group.


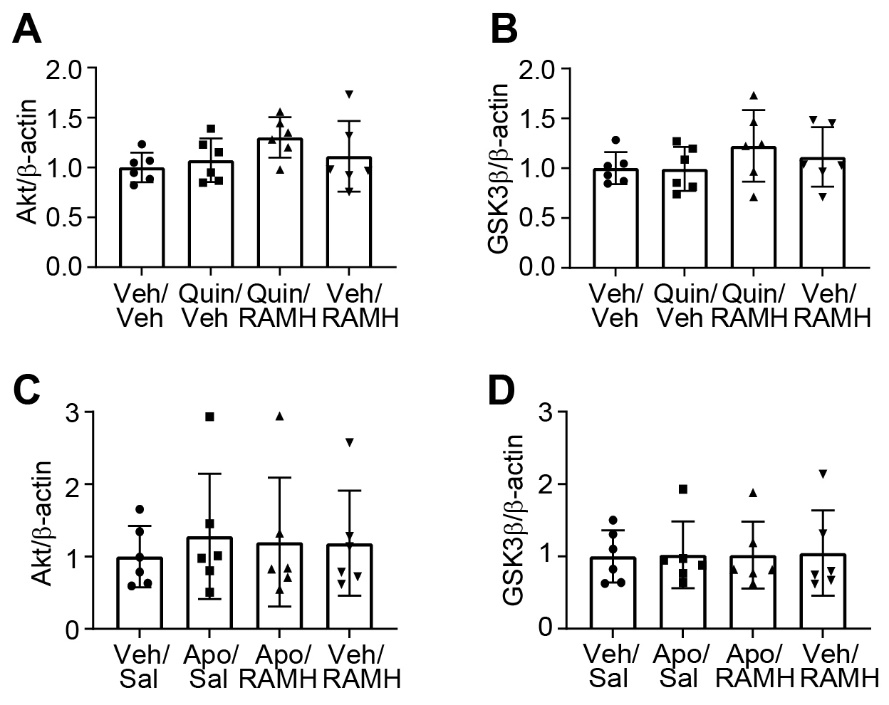


**Figure S9. No change in Akt/GSK3β total protein levels in striatal lysates after Quin-RAMH or Apo-RAMH treatment.** Male and female C57BL/6J mice received reserpine (2 mg/kg, s.c.) 20 h prior to drug administration. Mice received injections of saline (Sal) or RAMH (45 mg/kg, i.p.), followed by Sal or quinpirole (Quin, 0.5 mg/kg, i.p.), and sacrificed 30 min after treatment (***A* and *B***). Naïve C57BL/6J mice received injections of saline (Sal) or RAMH (45 mg/kg, i.p.), followed by vehicle (Veh) or apomorphine (Apo, 2 mg/kg, s.c.) and sacrificed 30 min after treatment (***C and D***). Protein levels were normalized to β-actin as a loading control. All values are expressed as mean ± SEM. No drug interactions or main effects of drugs were found. Statistical analysis was performed using two-way ANOVAs in GraphPad Prism 9. See **Table S2** for additional statistical analyses. n = 6 each group.


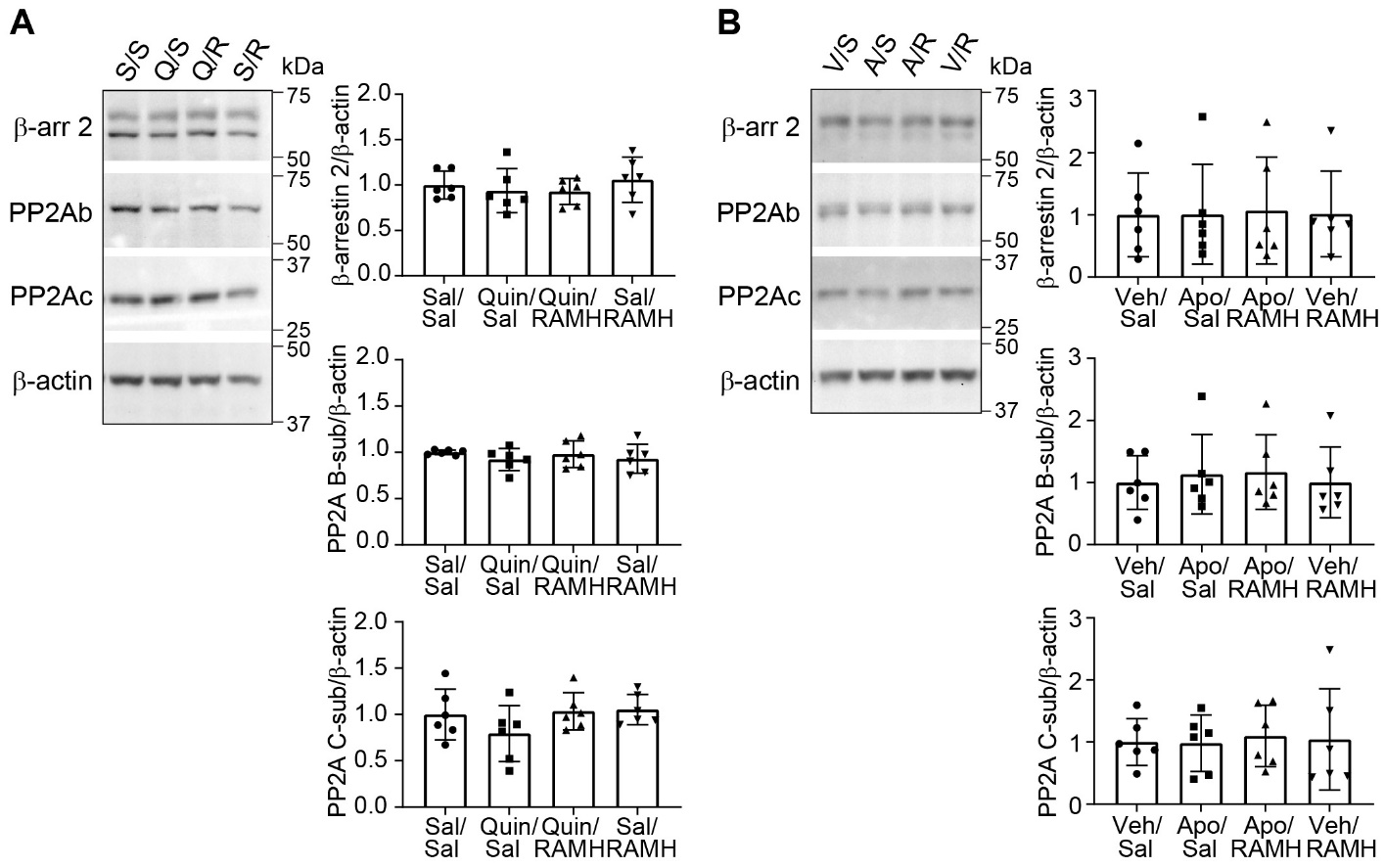


**Figure S10. No change in β-arrestin 2/PP2A protein levels in striatal lysates after Quin-RAMH or Apo-RAMH treatment.** Male and female C57BL/6J mice received reserpine (2 mg/kg, s.c.) 20 h prior to drug administration. Mice received injections of saline (Sal) or RAMH (45 mg/kg, i.p.), followed by Sal or quinpirole (Quin, 0.5 mg/kg, i.p.), and sacrificed 30 min after treatment (***A***). Naïve C57BL/6J mice received injections of saline (Sal) or RAMH (45 mg/kg, i.p.), followed by vehicle (Veh) or apomorphine (Apo, 2 mg/kg, s.c.) and sacrificed 30 min after treatment (***B***). Protein levels were normalized to β-actin as a loading control. All values are expressed as mean ± SEM. No drug interactions or main effects of drugs were found. Statistical analysis was performed using two-way ANOVAs in GraphPad Prism 9. See **Table S2** for additional statistical analyses. n = 6 each group. Representative images in ***A*** were reproduced from highlighted lanes in Fig. S11*A* (Fig. S11, *A* and *B* show the entire dataset for this experiment). The β-actin image is the same one used in Fig. 5*A*. Representative images in ***B*** were reproduced from highlighted lanes in Fig. S11*D* (Fig. S11, *C* and *D* show the entire dataset for this experiment). The β-actin image is the same one used in Fig. 5*B*.


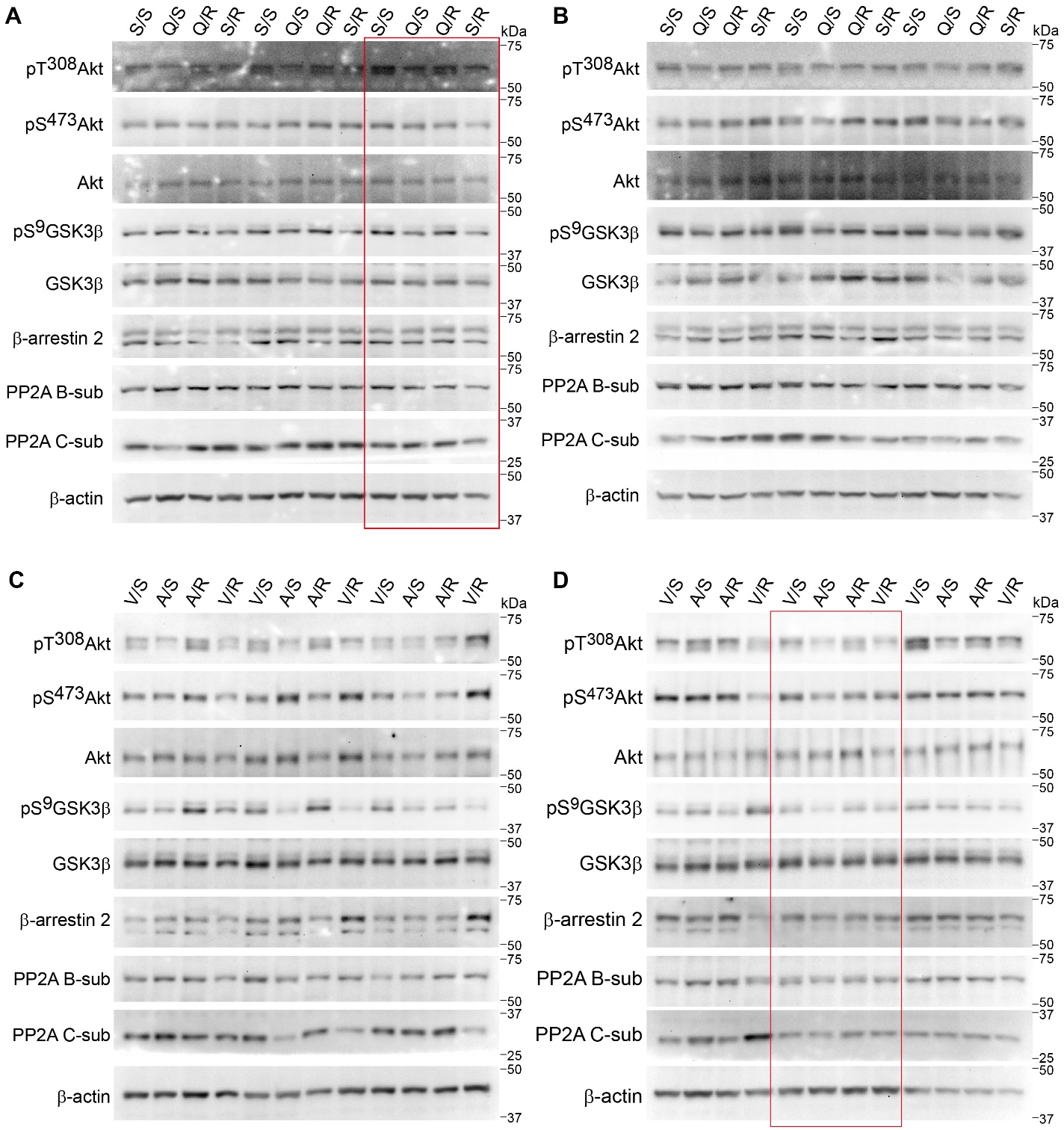


**Figure S11. Western blotting images related to Fig. 5.** Male and female C57BL/6J mice received reserpine (2 mg/kg, s.c.) 20 h prior to drug administration. Mice received injections of saline (Sal) or RAMH (45 mg/kg, i.p.), followed by Sal or quinpirole (Quin, 0.5 mg/kg, i.p.), and sacrificed 30 min after treatment (***A* and *B***). Naïve C57BL/6J mice received injections of saline (Sal) or RAMH (45 mg/kg, i.p.), followed by vehicle (Veh) or apomorphine (Apo, 2 mg/kg, s.c.) and sacrificed 30 min after treatment (***C* and *D***).Western blotting analysis of phosphorylation and total protein levels of the targets as indicated using specific antibodies (see **Table S1**). S: Saline; Q: quinpirole; A: apomorphine; V: vehicle; R: RAMH. n = 6 each group. Highlighted lanes were shown as representative images in **Fig. 5, *A* and *B***.


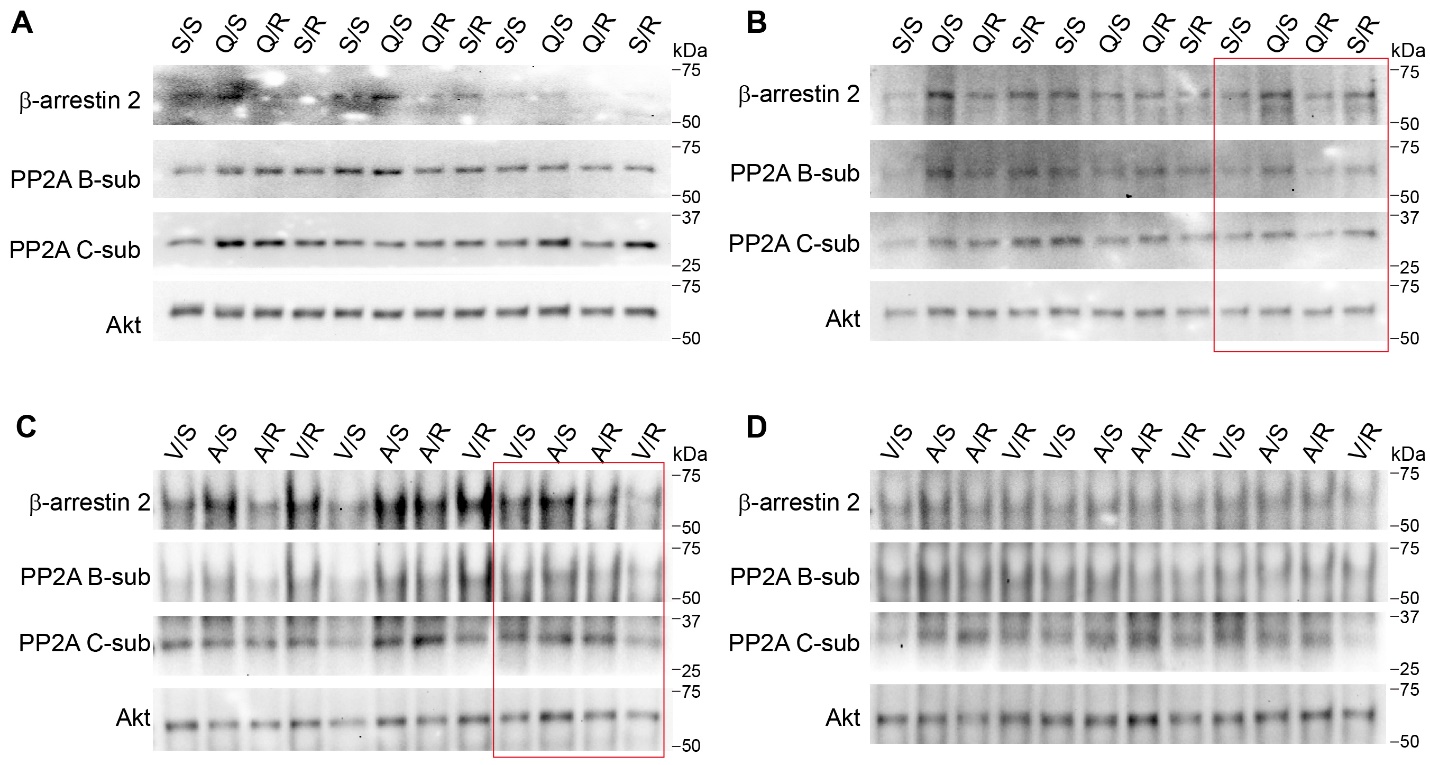


**Figure S12. Western blotting images related to Fig. 6.** Male and female C57BL/6J mice received reserpine (2 mg/kg, s.c.) 20 h prior to drug administration. Mice received injections of saline (Sal) or RAMH (45 mg/kg, i.p.), followed by Sal or quinpirole (Quin, 0.5 mg/kg, i.p.), and sacrificed 30 min after treatment (***A* and *B***). Naïve C57BL/6J mice received injections of saline (Sal) or RAMH (45 mg/kg, i.p.), followed by vehicle (Veh) or apomorphine (Apo, 2 mg/kg, s.c.) and sacrificed 30 min after treatment (***C* and *D***). Akt was immunoprecipitated from striatal lysates using anti-Akt antibody conjugated to Sepharose bead. Co-immunoprecipitation of β-arrestin 2 and PP2A subunits were probed using specific antibodies (see **Table S1**). S: Saline; Q: quinpirole; A: apomorphine; V: vehicle; R: RAMH. n = 6 each group. Highlighted lanes were shown as representative images in **Fig. 6, *A* and *B***.


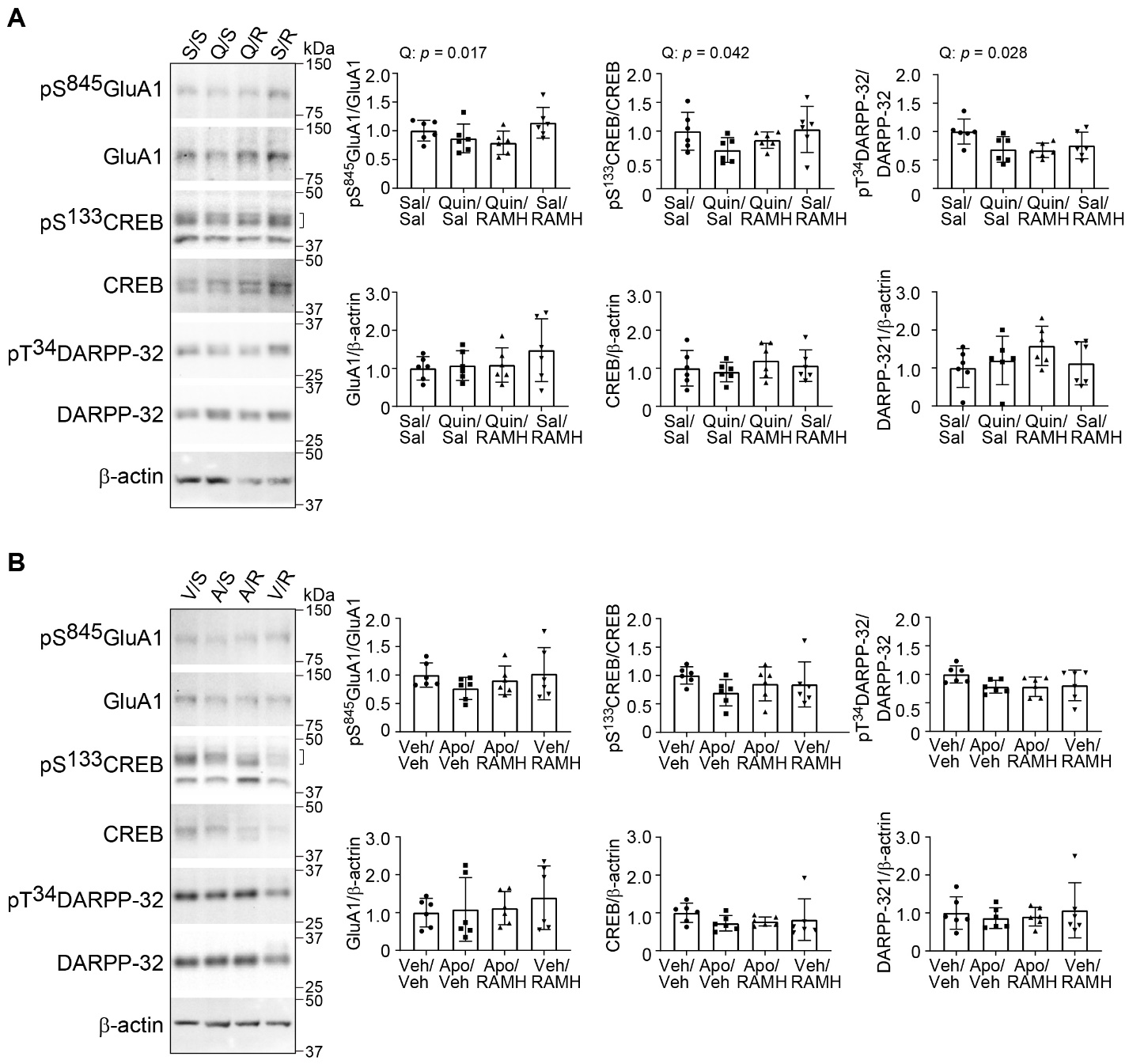


**Figure S13. H3R agonist treatment does not modulate the effects of D2R activation on cAMP-PKA signaling in mouse striatal lysates.** Male and female C57BL/6J mice received reserpine (2 mg/kg, s.c.) 20 h prior to drug administration. Mice received injections of saline (Sal) or RAMH (45 mg/kg, i.p.), followed by Sal or quinpirole (Quin, 0.5 mg/kg, i.p.), and sacrificed 30 min after treatment (*A*). Naïve C57BL/6J mice received injections of saline (Sal) or RAMH (45 mg/kg, i.p.), followed by vehicle (Veh) or apomorphine (Apo, 2 mg/kg, s.c.) and sacrificed 30 min after treatment (*B*). After treatment, phosphoprotein and total protein levels were assayed on Western blotting. Phosphorylation levels of GluA1, CREB and DARPP-32 were normalized to corresponding total protein levels. Pan-protein levels were normalized to β-actin as a loading control. ***A***, Phosphorylation and total protein levels of signal molecules in the striatal lysates after Quin-RAMH treatment. pS^845^ Akt, main effect of Quin: F (1, 20) = 6.74, *p* = 0.017, *η^2^_p_* = 0.252. pS^133^ CREB, Quin: F (1, 20) = 4.70, *p* = 0.042, *η^2^_p_* = 0.190. pT^34^ DARPP-32, Quin: F (1, 20) = 5.59, *p* = 0.028, *η^2^_p_* = 0.218. ***B***, Phosphorylation and total protein levels of signal molecules in the striatal lysates after Apo-RAMH treatment. All values are expressed as mean ± SD. Values were normalized to the corresponding Sal/Sal in A or Veh/Sal in B (each animal received two injections) group in each graph, respectively. Statistical analysis was performed using two-way ANOVAs in GraphPad Prism 9. See **Table S2** for additional statistical analyses. n = 6 each group. Representative images in ***A*** were reproduced from highlighted lanes in Fig. S14*A* (Fig. S14, *A* and *B* show the entire dataset for this experiment). Representative images in ***B*** were reproduced from highlighted lanes in Fig. S14*C* (Fig. S14, *C* and *D* show the entire dataset for this experiment).


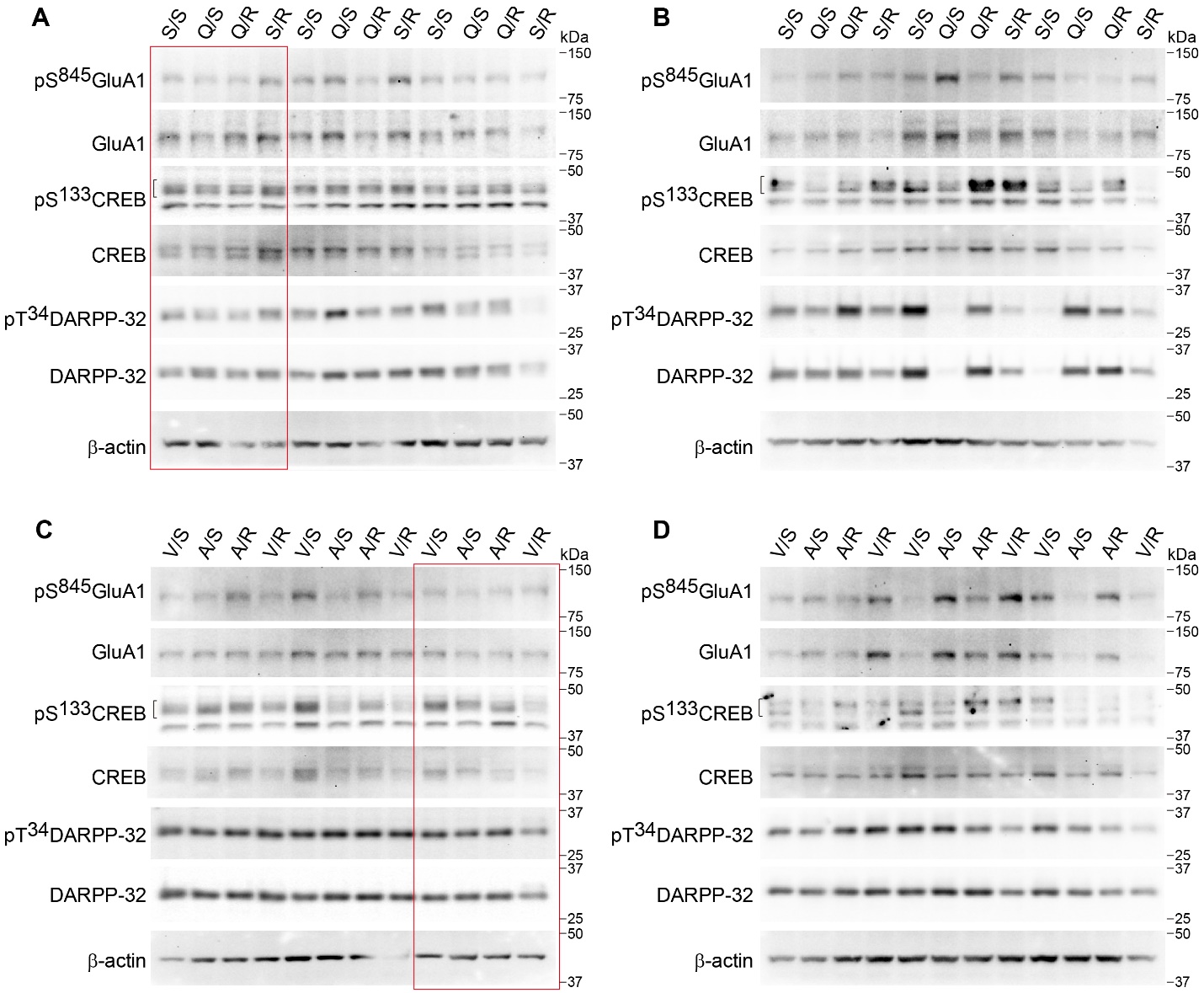


**Figure S14. Western blotting images related to Fig. S13.** Male and female C57BL/6J mice received reserpine (2 mg/kg, s.c.) 20 h prior to drug administration. Mice received injections of saline (Sal) or RAMH (45 mg/kg, i.p.), followed by Sal or quinpirole (Quin, 0.5 mg/kg, i.p.), and sacrificed 30 min after treatment (***A* and *B***). Naïve C57BL/6J mice received injections of saline (Sal) or RAMH (45 mg/kg, i.p.), followed by vehicle (Veh) or apomorphine (Apo, 2 mg/kg, s.c.) and sacrificed 30 min after treatment (***C* and *D***).Western blotting analysis of phosphorylation and total protein levels of the targets as indicated using specific antibodies (see **Table S1**). S: Saline; Q: quinpirole; A: apomorphine; V: vehicle; R: RAMH. n = 6 each group. Highlighted lanes were shown as representative images in **Fig. S13, *A* and *B***. Images in *A* and *B* and those in Fig. S16, *A* and *B* were from the same set of membranes, therefore they shared the same load control β-actin. Similarly, images in *C* and *D* and those in Fig. S16, *C* and *D* were from the same set of membranes, therefore they shared the same load control β-actin.


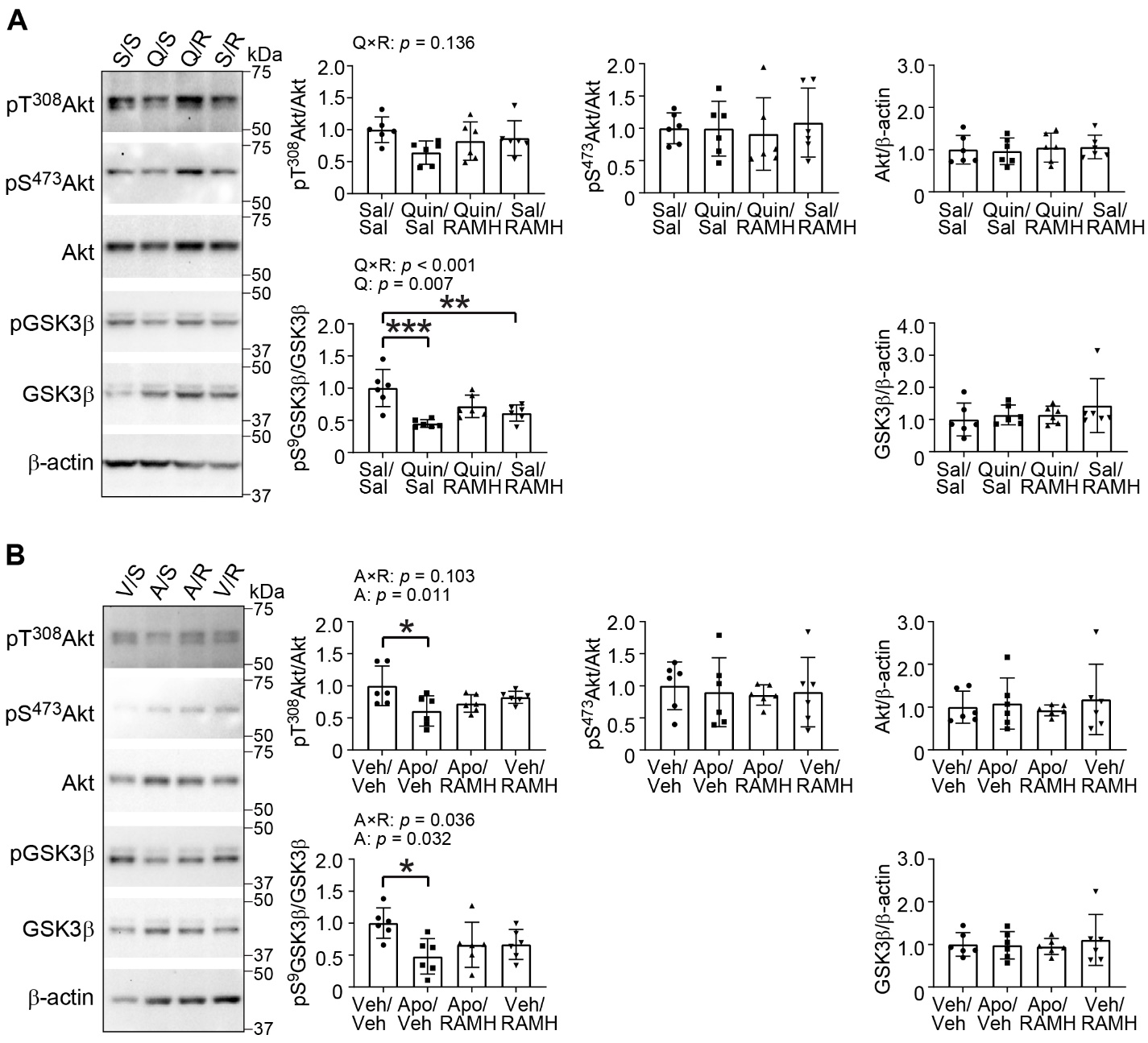


**Figure S15. H3R agonist treatment modulates the effects of D2R activation on Akt-GSKβ signaling in mouse striatal lysates.** Male and female C57BL/6J mice received reserpine (2 mg/kg, s.c.) 20 h prior to drug administration. Mice received injections of saline (Sal) or RAMH (45 mg/kg, i.p.), followed by Sal or quinpirole (Quin, 0.5 mg/kg, i.p.), and sacrificed 30 min after treatment (*A*). Naïve C57BL/6J mice received injections of saline (Sal) or RAMH (45 mg/kg, i.p.), followed by vehicle (Veh) or apomorphine (Apo, 2 mg/kg, s.c.) and sacrificed 30 min after treatment (*B*). After treatment, phosphoprotein and total protein levels were assayed on Western blotting. Phosphorylation levels of Akt and GSK3β were normalized to corresponding total protein levels. Pan-protein levels were normalized to β-actin as a loading control. ***A***, Phosphorylation and total protein levels of signal molecules in the striatal lysates after Quin-RAMH treatment. pT^308^ Akt, Quin × RAMH interaction: F (1, 20) = 2.41, p = 0.136; main effect of Quin: F (1, 20) = 3.99, *p* = 0.060. pS^9^ GSK3β, Quin × RAMH interaction: F (1, 20) = 19.50, *p* = 0.0003, *η^2^_p_* = 0.494; main effect of Quin: F (1, 20) = 8.98, *p* = 0.007, *η^2^_p_* = 0.310. Quin/Sal vs Sal/Sal: p = 0.0002; Sal/RAMH vs Sal/Sal: p = 0.007. ***B***, Phosphorylation and total protein levels of signal molecules in the striatal lysates after Apo-RAMH treatment. pT^308^ Akt, Apo × RAMH interaction: F (1, 20) = 2.92, *p* = 0.103. Apo/Sal vs Veh/Sal: p = 0.021. pS^9^ GSK3β, Apo × RAMH interaction: F (1, 20) = 5.05, *p* = 0.036, *η^2^_p_* = 0.202; main effect of Apo: F (1, 20) = 5.30, *p* = 0.032, *η^2^_p_* = 0.210. Apo/Sal vs Veh/Sal: p = 0.021. All values are expressed as mean ± SD. Values were normalized to the corresponding Sal/Sal in A or Veh/Sal in B (each animal received two injections) group in each graph, respectively. Statistical analysis was performed using two-way ANOVAs in GraphPad Prism 9. See **Table S2** for additional statistical analyses. Where significant drug interactions or main effects were found, multiple comparisons were conducted using post hoc Tukey test. **p* < 0.05, ***p* < 0.01, ****p* < 0.001, n = 6 each group. Representative images in ***A*** were reproduced from highlighted lanes in Fig. S16*B* (Fig. S16, *A* and *B* show the entire dataset for this experiment). Representative images in ***B*** were reproduced from highlighted lanes in Fig. S16*C* (Fig. S16, *C* and *D* show the entire dataset for this experiment).


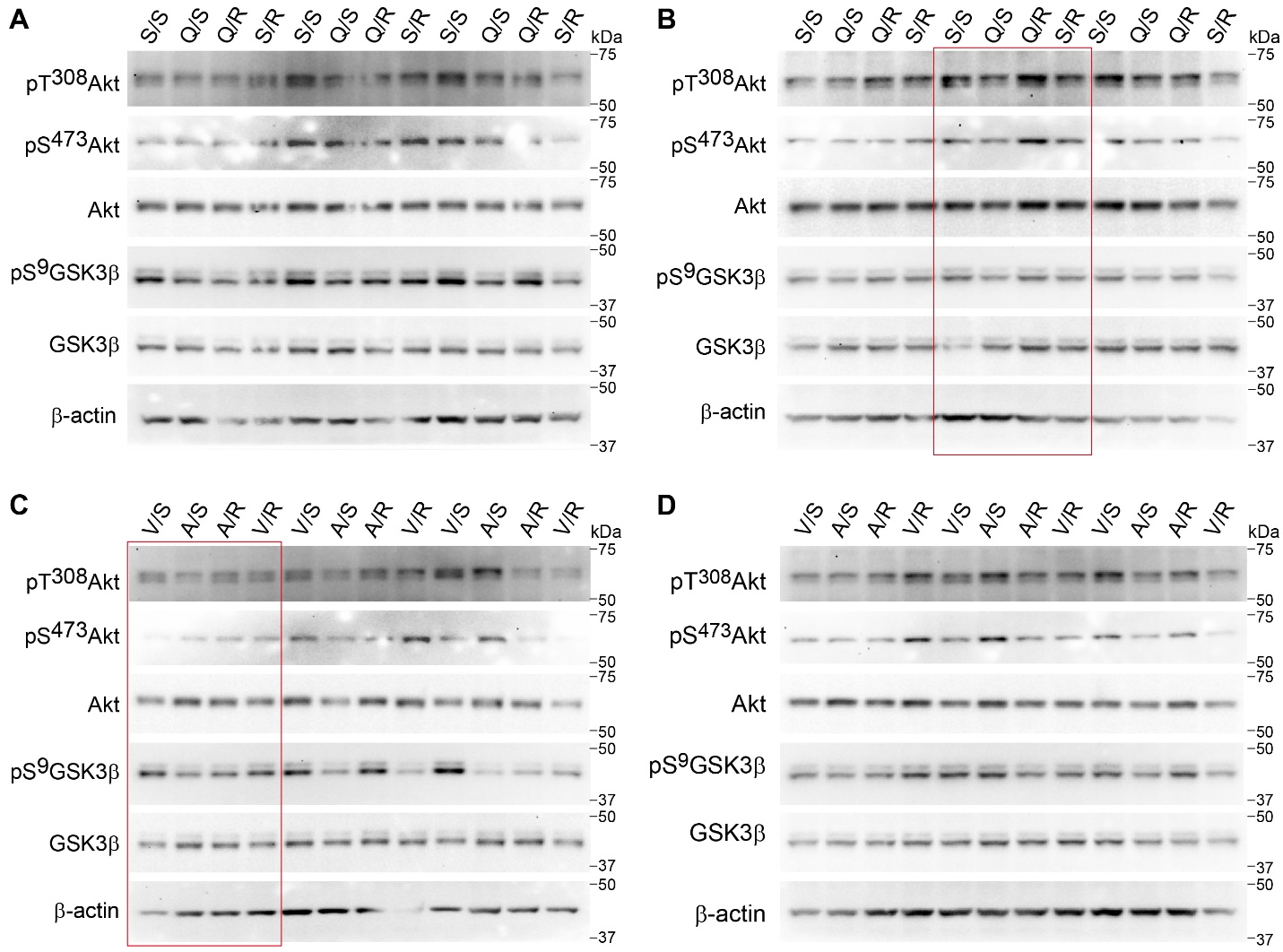


**Figure S16. Western blotting images related to Fig. S15.** Male and female C57BL/6J mice received reserpine (2 mg/kg, s.c.) 20 h prior to drug administration. Mice received injections of saline (Sal) or RAMH (45 mg/kg, i.p.), followed by Sal or quinpirole (Quin, 0.5 mg/kg, i.p.), and sacrificed 30 min after treatment (***A* and *B***). Naïve C57BL/6J mice received injections of saline (Sal) or RAMH (45 mg/kg, i.p.), followed by vehicle (Veh) or apomorphine (Apo, 2 mg/kg, s.c.) and sacrificed 30 min after treatment (***C* and *D***).Western blotting analysis of phosphorylation and total protein levels of the targets as indicated using specific antibodies (see **Table S1**). S: Saline; Q: quinpirole; A: apomorphine; V: vehicle; R: RAMH. n = 6 each group. Highlighted lanes were shown as representative images in **Fig. S15, *A* and *B***. Images in *A* and *B* and those in Fig. S14, *A* and *B* were from the same set of membranes, therefore they shared the same load control β-actin. Similarly, images in *C* and *D* and those in Fig. S14, *C* and *D* were from the same set of membranes, therefore they shared the same load control β-actin.


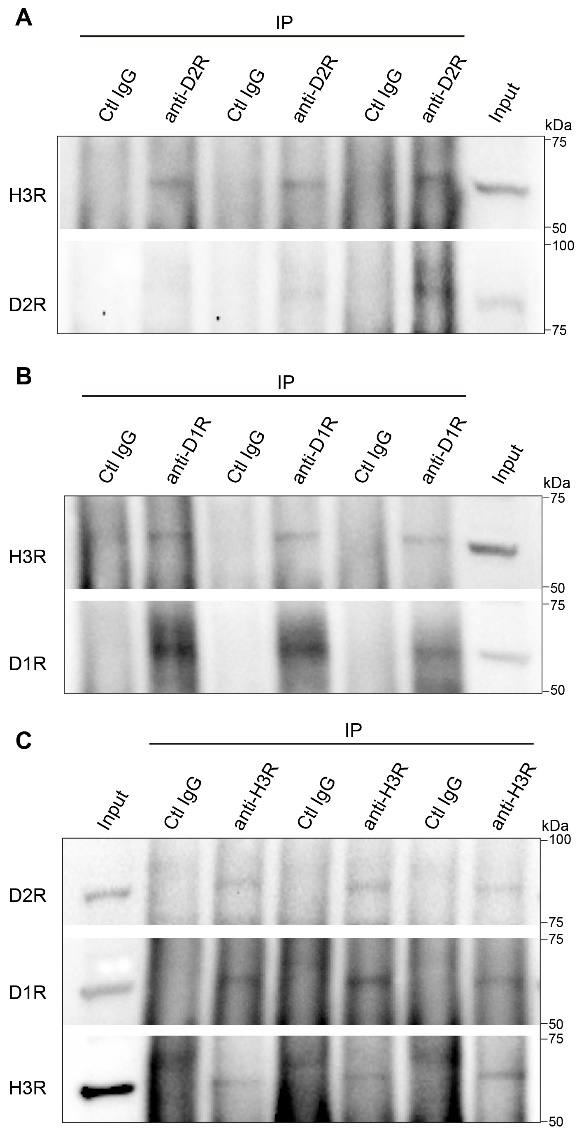


**Figure S17. Western blotting images related to Fig. 7, *A*-*C*.** Striatal lysates from naïve Male and female C57BL/6J mice were used for immunoprecipitation with anti-D2R antibody (***A***), anti-D1R antibody (***B***) or anti-H3R antibody (***C***). Isotype IgGs were used as negative controls. Co-immunoprecipitation of H3R by anti-D2R antibody (***A***) and anti-D1R antibody (***B****)*, or co-immunoprecipitation of D2R and D1R by anti-H3R antibody (***C***) were assayed on Western blotting using specific antibodies (see **Table S1**). Images show additional biological replicates (n = 3 each group) related to **Fig. 7, *A*-*C***.

.


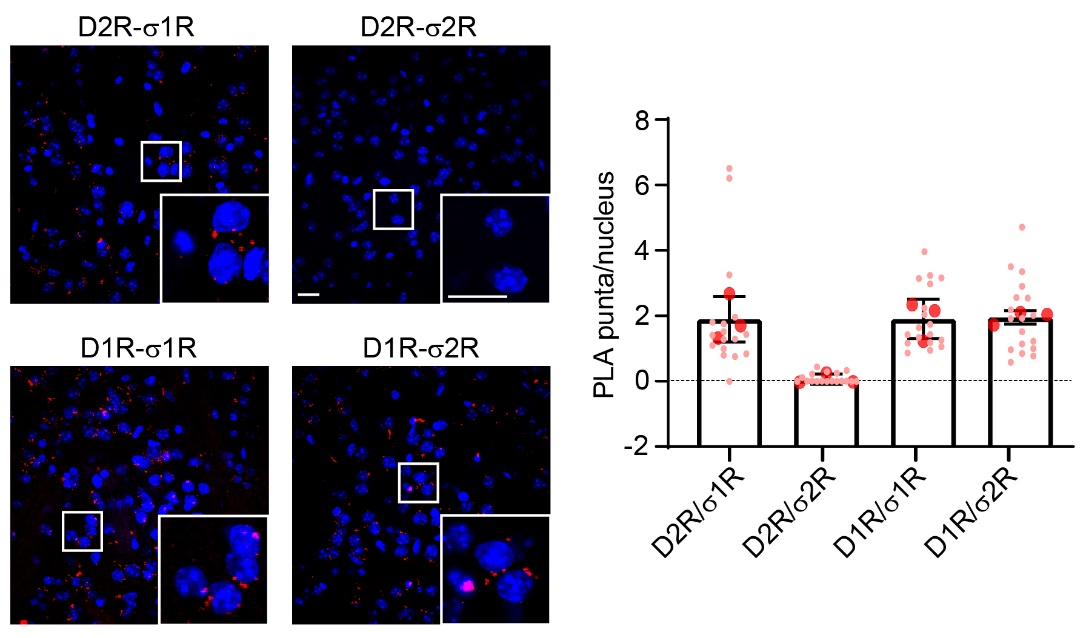


**Figure S18. Additional control groups for promixity ligation assay related to Figure 6D.** Striatal sections were immunostained using D2R-σ1R, D2R-σ2R, D1R-σ1R and D1R-σ2R antibody pairs. Positive PLA signal (when antibody pair labeled proteins in close proximity) was visualized as red puncta around cell nuclei counterstained by DAPI (blue). Scale bar: 20 μm. Average number of red puncta per nucleus was used as a measure of PLA signal. All values are expressed as mean ± SEM. n = 3 each group.

**Table S1. Antibodies used in this study**

| **Antibody** | **Host** | **Dilution** | **Source** | **Catalog#** | **Application** |
| --- | --- | --- | --- | --- | --- |
| Anti-FLAG | mouse | 1:1000 | Sigma | F1804 | IHC |
| Anti-Myc | goat | 1:1000 | Abcam | ab9132 | IHC |
| Anti-phospho-Akt (Thr^308^) | rabbit | 1:1000 | Cell Signaling | 2965S | IHC |
| Anti-phospho-Akt (Thr^308^) | rabbit | 1:500 | Cell Signaling | 13038S | WB |
| Anti-phospho-Akt (Ser^473^) | rabbit | 1:1000 | Cell Signaling | 4060S | WB |
| Anti-Akt (pan) | rabbit | 1:2000 | Cell Signaling | 4691S | WB |
| Anti-Akt (pan) Sepharose conjugate | mouse | 1:100 | Cell Signaling | 3653S | IP |
| Anti-phospho-GSK3β (Ser^9^) | rabbit | 1:100  1:500 | Cell Signaling | 9323S | IHC,  WB |
| Anti-GSK3β | rabbit | 1:5000 | Cell Signaling | 9315S | WB |
| Anti-phospho- phospho-MSK1 (Thr^581^) | rabbit | 1:100 | Cell Signaling | 9595S | IHC |
| Anti-phospho-rpS6 (Ser^235/236^) | rabbit | 1:800 | Cell Signaling | 4858S | IHC |
| Anti-phospho-rpS6 (Ser^240/244^) | rabbit | 1:2000 | Cell Signaling | 5364S | IHC |
| Anti-PP2A B subunit | rabbit | 1:1000 | Cell Signaling | 4953S | WB |
| Anti-PP2A C subunit | rabbit | 1:1000 | Cell Signaling | 2038S | WB |
| Anti-β-arrestin 2 | rabbit | 1:1000 | Novus Biologicals | NB300-587 | WB |
| Anti-phospho-GluA1 (Ser^845^) | rabbit | 1:1000 | Cell Signaling | 8084S | WB |
| Anti-phospho-GluA1 | rabbit | 1:1000 | Millipore | 05-855R | WB |
| Anti-phospho-CREB (Ser^133^) | rabbit | 1:500 | Cell Signaling | 9198S | WB |
| Anti-phospho-CREB | rabbit | 1:1000 | Cell Signaling | 9197S | WB |
| Anti-phospho-DARPP-32 (Thr^34^) | rabbit | 1:500 | Abcam | ab51076 | WB |
| Anti-phospho-DARPP-32 | rabbit | 1:2000 | Millipore | AB10518 | WB |
| Anti-β-actin | mouse | 1:10,000 | Santa Cruz Biotechnology | sc-47778 | WB |
| Anti-H3R | mouse | 1:100 | Alpha Diagnostic International | H3R31-A | IHC |
| Anti-H3R | goat | 1:1000, 1:100 | Santa Cruz | sc-17921 | WB,  IP |
| Anti-D1R | goat | 1:500  1:1000  1:100 | Frontier Institute Co. Ltd | D1R-Go-Af1000 | IHC, PLA  WB  IP |
| Anti-D2R | guinea pig | 1:500 | Frontier Institute Co. Ltd | D2R-GP-Af500 | IHC, PLA |
| Anti-D2R | rabbit | 1:500  1:100 | Frontier Institute Co. Ltd | D2R-Rb-Af960 | WB  IP |
| Anti-σ1R | rabbit | 1:100 | Novus Biologicals | NBP1-82479 | PLA |
| Anti-σ2R | rabbit | 1:100 | Novus Biologicals | NBP1-30436 | PLA |
| Alexa Fluor 405 donkey anti-mouse | donkey | 1:800 | Thermo Fisher Scientific | A48257 | IHC |
| Alexa Fluor 488 donkey anti-rabbit | donkey | 1:800 | Thermo Fisher Scientific | A21206 | IHC |
| Alexa Fluor 594 donkey anti-mouse | donkey | 1:800 | Thermo Fisher Scientific | A21203 | IHC |
| Alexa Fluor 594 donkey anti-goat | donkey | 1:800 | Thermo Fisher Scientific | A11058 | IHC |
| Alexa Fluor 633 donkey anti-goat | donkey | 1:800 | Thermo Fisher Scientific | A21082 | IHC |
| Anti-rabbit IgG (H+L)-HRP conjugate | goat | 1:10,000 | Thermo Fisher Scientific | 31460 | WB |
| Anti-mouse IgG (H+L)-HRP conjugate | goat | 1:10,000 | Thermo Fisher Scientific | 31430 | WB |
| Anti-goat IgG (H+L)-HRP conjugate | rabbit | 1:10,000 | Thermo Fisher Scientific | 31402 | WB |

**Table S3. Hrh3 expression in scRNAseq datasets**

| **Cluster^1^** | **Munoz-Manchado et al., 2018^2^** | **Zeisel et al., 2018^2^** | **Gokce et al., 2016^2,3^** | **Saunders et al., 2018^2,4^** |
| --- | --- | --- | --- | --- |
| D1-SPNs | 0.80 | 0.27 | 3.66 | 2.56 |
| D2-SPNs | 1.45 | 0.29 | 3.14 | 2.48 |
| ChAT-INs | 0.10 | 0.23 | 3.24 | 1.79 |
| PV-INs | 3.00 | 0.42 |  | 2.56 |
| TH-INs | 0.45 | 0.14 |  |  |
| SST-INs | 0.90 | 0.15 |  | 1.39 |
| Microglia | 0.07 | 0.00 | 0.00 | 1.39 |
| Astrocytes | 0.15 | 0.01 | 0.04 | 0.69 |
| Oligodendrocytes | 0.40 | 0.00 | 0.00 | 0.69 |
| OPCs | 0.00 | 0.01 | 0.24 | 0.69 |
| Endothelial | 0.31 | 0.00 | 0.05 | 0.69 |
| Vascular Smooth Muscle | 0.18 | 0.00 | N/A | N/A |

**^1^**Cluster names correspond to those used in Munoz-Manchado et al., 2018. SPNs: spiny projection neurons; INs: interneurons; ChAT: choline acetyltransferase; PV: parvalbumin; TH: tyrosine hydroxylase; SST: Somatostatin; OPC: oligodendrocyte precursor cells.

^2^Values were obtained in datasets from each study, in which different quantification/normalization procedures were used. N/A: no values available.

^3^Value represents the expression level in mixed interneurons. Subtypes were not distinguished due to not enough cells retrieved.

^4^Value represents the expression level in PV- or TH-interneurons. Subtypes were not distinguished.

**References**

1. Gokce, O., Stanley, G. M., Treutlein, B., Neff, N. F., Camp, J. G., Malenka, R. C., Rothwell, P. E., Fuccillo, M. V., Südhof, T. C., and Quake, S. R. (2016) Cellular Taxonomy of the Mouse Striatum as Revealed by Single-Cell RNA-Seq. *Cell Rep* **16**, 1126-1137

2. Muñoz-Manchado, A. B., Bengtsson Gonzales, C., Zeisel, A., Munguba, H., Bekkouche, B., Skene, N. G., Lönnerberg, P., Ryge, J., Harris, K. D., Linnarsson, S., and Hjerling-Leffler, J. (2018) Diversity of Interneurons in the Dorsal Striatum Revealed by Single-Cell RNA Sequencing and PatchSeq. *Cell Rep* **24**, 2179-2190.e2177

3. Zeisel, A., Hochgerner, H., Lönnerberg, P., Johnsson, A., Memic, F., van der Zwan, J., Häring, M., Braun, E., Borm, L. E., La Manno, G., Codeluppi, S., Furlan, A., Lee, K., Skene, N., Harris, K. D., Hjerling-Leffler, J., Arenas, E., Ernfors, P., Marklund, U., and Linnarsson, S. (2018) Molecular Architecture of the Mouse Nervous System. *Cell* **174**, 999-1014.e1022

4. Saunders, A., Macosko, E. Z., Wysoker, A., Goldman, M., Krienen, F. M., de Rivera, H., Bien, E., Baum, M., Bortolin, L., Wang, S., Goeva, A., Nemesh, J., Kamitaki, N., Brumbaugh, S., Kulp, D., and McCarroll, S. A. (2018) Molecular Diversity and Specializations among the Cells of the Adult Mouse Brain. *Cell* **174**, 1015-1030.e1016
